# Grid Cells Form a Global Representation of Connected Environments

**DOI:** 10.1016/j.cub.2015.02.037

**Published:** 2015-05-04

**Authors:** Francis Carpenter, Daniel Manson, Kate Jeffery, Neil Burgess, Caswell Barry

**Affiliations:** 1 Institute of Neurology, UCL, Queen Square, London WC1N 3BG, UK; 2 Research Department of Cell and Developmental Biology, UCL, Gower Street, London WC1E 6BT, UK; 3 Centre for Mathematics and Physics in the Life Sciences and Experimental Biology, UCL, Gower Place, London WC1E 6BT, UK; 4 Institute of Behavioural Neuroscience, UCL, Bedford Way, London WC1H 0AP, UK; 5 Institute of Cognitive Neuroscience, UCL, Queen Square, London WC1N 3AR, UK

## Abstract

The firing patterns of grid cells in medial entorhinal cortex (mEC) and associated brain areas form triangular arrays that tessellate the environment [1, 2] and maintain constant spatial offsets to each other between environments [3, 4]. These cells are thought to provide an efficient metric for navigation in large-scale space [5–8]. However, an accurate and universal metric requires grid cell firing patterns to uniformly cover the space to be navigated, in contrast to recent demonstrations that environmental features such as boundaries can distort [9–11] and fragment [12] grid patterns. To establish whether grid firing is determined by local environmental cues, or provides a coherent global representation, we recorded mEC grid cells in rats foraging in an environment containing two perceptually identical compartments connected via a corridor. During initial exposures to the multicompartment environment, grid firing patterns were dominated by local environmental cues, replicating between the two compartments. However, with prolonged experience, grid cell firing patterns formed a single, continuous representation that spanned both compartments. Thus, we provide the first evidence that in a complex environment, grid cell firing can form the coherent global pattern necessary for them to act as a metric capable of supporting large-scale spatial navigation.

## Highlights

• Grid cells were recorded in connected, perceptually identical compartments
• Initial grid firing patterns replicated between the two compartments
• With experience, a single, continuous grid pattern spanning both compartments formed
• This globally coherent pattern allows grid cells to act as a spatial metric

## Graphical Abstract

**Figure:**
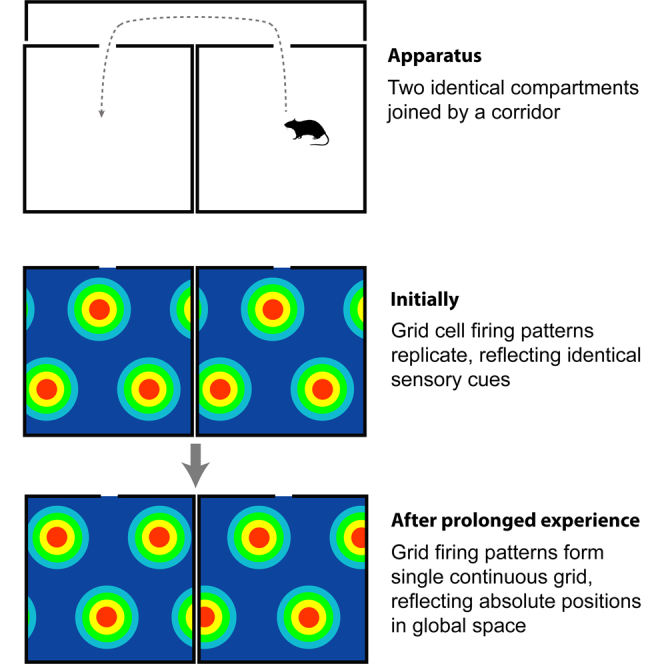

## Teaser

Grid cells are thought to provide an efficient metric for spatial navigation. However, this requires regular, continuous grid firing patterns across the environment. Carpenter et al. show that grid patterns are initially determined by sensory cues but self-correct with experience to form the globally coherent pattern required of a spatial metric.

## Results

**Figure 1:**
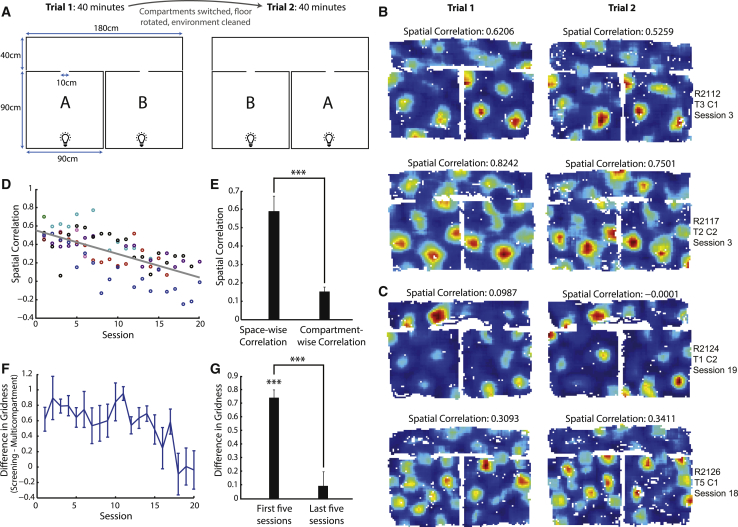
With Increasing Experience, Grid Cell Firing Patterns Show Reduced Representation Similarity between Compartments and Increased Regularity (A) Schematic representation of the multicompartment environment and protocol for each recording session. (B and C) Example firing rate maps. The left and right rate maps in each row are the same cell recorded in trial 1 and trial 2, respectively. Hotter colors indicate higher firing rates; unvisited bins are white. The correlation values above each plot are the spatial correlations of firing rates between the two compartments. (B) Example grid cells recorded during early exposures to the multicompartment environment, where firing fields replicated between compartments. (C) Example grid cells from late recording sessions, where firing patterns distinguished the compartments. (D) Spatial correlations of grid cell firing rates between the compartments as a function of the animals’ experience of the environment. Each data point represents the average correlation across all cells from one animal in one session, with different animals plotted in different colors. (E) Spatial correlations between grid cell firing in equivalent absolute locations in successive trials (“space-wise correlation”: e.g., compartment A trial 1 versus compartment B trial 2) or between equivalent locations within the same physical compartment in successive trials (“compartment-wise correlation”: e.g., compartment A trial 1 versus compartment A trial 2), showing mean + SEM for cells in the last five sessions. (F) The difference in gridness of firing patterns between the familiar square screening environment and the average of the gridness in each compartment (screening gridness − multicompartment gridness) as a function of experience. Plotted values are mean ± SEM across all cells recorded in each session. (G) Difference in gridness (screening gridness − multicompartment gridness) in the first and last five sessions, showing mean and SEM; ^∗∗∗^p < 0.001.

We investigated whether grid cell firing patterns are determined by local sensory cues or whether they provide a coherent global representation of space by recording from 85 medial entorhinal cortex (mEC) grid cells in eight rats as they foraged within an environment containing two perceptually identical compartments connected via a corridor (Figure 1A). The environment was painted matte black and lit only by single lights on the south wall of each compartment. Black curtains encircled the environment to reduce the availability of distal cues. Each recording session consisted of two 40-min trials, with the floor of the environment rotated and the positions of the compartments swapped between trials to control unidentified sensory cues distinguishing the compartments. The environment therefore closely matched environments in which perceptual consistency between adjacent compartments has been shown to cause place cell firing fields to replicate [13–15]. We hypothesized that if grid cell representations are dominated by sensory cues, their firing should replicate between the two compartments. Conversely, if grid cell activity is determined by the global spatial features of the environment, their firing patterns should distinguish the two compartments due to their different absolute positions in space.

During early sessions, periodic firing patterns typical of grid cells were present in the environment and were replicated between the two compartments (Figure 1B). However, with increasing experience, the similarity of the representations between the two compartments decreased (Figure 1C), apparent in a negative correlation between the session number and the spatial correlation of firing rates between the two compartments (r = −0.6674, r^2^ = 0.4455, p = 1.669 × 10^−12^; Figure 1D). The decrease in representation similarity across sessions was accompanied by an increase in hexagonal regularity of grid patterns within the compartments (Figure 1F). In the first five sessions, but not the last five sessions, gridness in the screening environment was greater than in the multicompartment environment (one-sample t tests, t_41_ = 11.46, p = 2.328 × 10^−14^ and t_25_ = 0.8519, p = 0.4024, respectively; Figure 1G), with the difference in gridness greater in the first than the last five sessions (unpaired t test, t_66_ = 5.279, p = 1.443 × 10^−6^).

To eliminate the possibility that unidentified local cues allowed disambiguation of the two compartments, we verified that firing was stable in global space and did not track the physical compartments when their positions were switched between trials. Specifically, in the last five sessions, the inter-trial spatial correlation between compartments in the same location was greater than the inter-trial spatial correlation between the same physical compartments in their new positions (paired t test, t_20_ = 6.560, p = 2.160 × 10^−6^; Figure 1E).

In contrast to grid cells, head direction cells continued to show the same directional tuning in the two compartments, regardless of experience (Figures S1A–S1C). The firing of a single border cell recorded in a late session also replicated between the two compartments (Figures S1D–S1F).

To determine whether grid firing in the two compartments predominantly reflected a local or global reference frame, we fitted ideal grid patterns to the recorded firing rate maps according to three models (see Supplemental Experimental Procedures for details). The grids were first fit by the “independent” model, in which grid phase was allowed to vary freely between the two compartments, while orientation and scale were required to be consistent (see Figures S2A–S2D). The independent fit represented the best possible fit of an ideal grid pattern to the data and was used to exclude grids too irregular to be well fit by any model, a necessary step given the reduced gridness seen in the multicompartment environment, particularly during early sessions (Figure 1F). The independent fit also determined the scale and orientation used in the “local” and “global” models. In the local model, the fitted grid had the same phase in each compartment, such that the firing fields replicated. The global model required the phase to be continuous across the compartments and so formed a single grid spanning the two. Local and global fits were normalized by the independent fit to allow comparison across cells. Firing patterns in the corridor were spatially stable and “grid-like”—consisting of peaks and troughs in firing. However, they were significantly less regular than firing patterns in the compartments and were discarded from further analyses (Figures S3A–S3C). The irregularity was likely caused by the stereotyped behavior displayed by rats in the corridor (Figures S3D–S3F) and is consistent with past recordings of grid cells in linear environments [16, 17].

**Figure 2:**
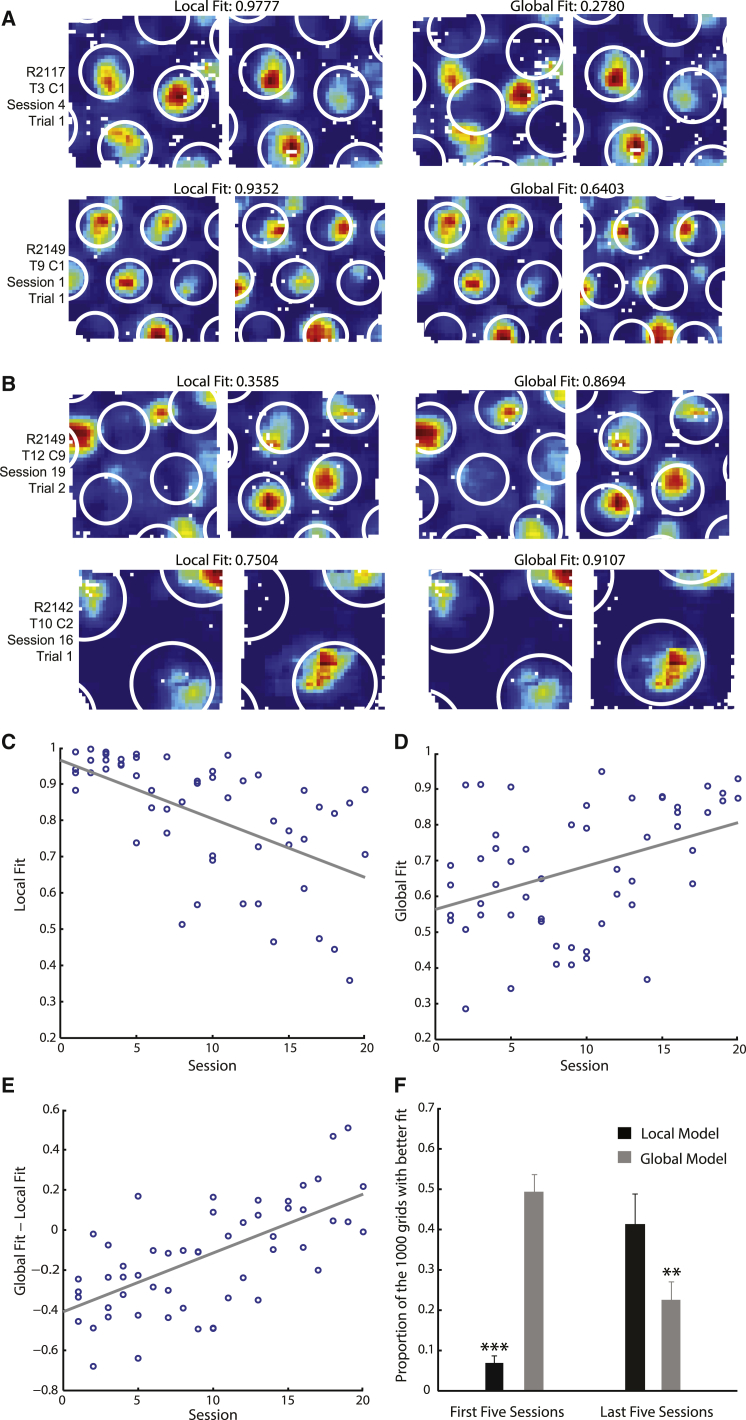
Grid Cell Firing Patterns Transition from a Local to a Global Representation with Increasing Experience (A and B) Fits of local and global models to grid cell firing patterns in the two compartments. The local model was an ideal grid constrained to replicate between the two compartments, whereas the global model was a single continuous grid spanning both compartments. Each row is one cell in one trial: the underlying rate maps in the left and right columns are the same. The white rings overlaid indicate the best fitting local and global models in the left and right columns, respectively. Fit values show the spatial correlations between the local or global models and the data, normalized by the independent model’s fit. (A) Examples of grid cells recorded during early sessions, where the local model best fit the data. (B) Example grids recorded during late sessions, where the global model best fit the data. (C and D) The fit between grid cell firing patterns and ideal local and global grids, respectively, as a function of experience of the environment. (E) The difference in the fit (global fit − local fit) between the global and local models across sessions. In (C), (D), and (E), each data point represents the average fit for all cells with an independent fit >0.45, recorded from one animal in one session. (F) The proportion of 1,000 ideal grids, with random phase offsets between compartments, with a better fit to the data than the local or global models. Values are mean + SEM across all cells with an independent fit >0.45 in the first or last five sessions. Wilcoxon signed-rank tests (WSRTs) compare observed values to an expected median of 0.5. ^∗∗^p < 0.01; ^∗∗∗^p < 0.001.

During early exposures, grid cell firing in the two compartments was best described by the local model (Figure 2A). However, with increasing experience, the local model’s fit to the data decreased (r *= −*0.5913, r^2^ = 0.3496, p = 2.503 × 10^−6^; Figure 2C). In contrast to the local model, the fit to the global model increased with experience, showing a positive correlation (r = 0.4187, r^2^ = 0.1753, p = 0.0016; Figures 2B and 2D). A two-way ANOVA revealed an interaction between experience of the environment and the goodness of fit of the two models (session × model, F_(19,68)_ = 1.89, p = 0.0293; Figure 2E). We assessed whether in the first and last five sessions the local or global models fitted the data significantly better than would be expected under the null hypothesis of no particular phase relationship between the grids in each compartment. Specifically, each recorded rate map was fitted by 1,000 ideal grids with random phase offsets between the two compartments. For each cell/session conjunction with an independent fit >0.45, we then calculated the proportion of the 1,000 grids with random phase offsets which achieved a better fit than the local and global models. If no particular phase relationship between the grids in each compartment existed, the local and global models would on average fall in the middle of the distribution of the randomly offset grids. In contrast, in the first five sessions, the proportion of the 1,000 grids with a better fit than the local model was significantly lower than 0.5 (Wilcoxon signed-rank test [WSRT], z = −3.724, p = 1.964 × 10^−4^; Figure 2F). However, in the last five sessions, the local model no longer fit the data better than would be expected by chance (WSRT, p *=* 0.6377; Figure 2F). Conversely, in the last five sessions, but not the first five sessions, the global model’s fit to the data was significantly better than expected from the null distribution (WSRTs, p *=* 0.0019 and z = −0.1089, p = 0.9133; Figure 2F). It is important to note that local and global representations are not mutually exclusive: grid patterns can be both identical in the two compartments and continuous across them both. As such, one would not necessarily expect consistently low local fits during late sessions or consistently low global fits during early sessions.

An independent analysis of the difference between observed grid phase in the right-hand compartment and that predicted from the left-hand compartment if firing formed a local or global representation confirmed a transition from a local to a global firing pattern (Figures S2G and S2H).

**Figure 3:**
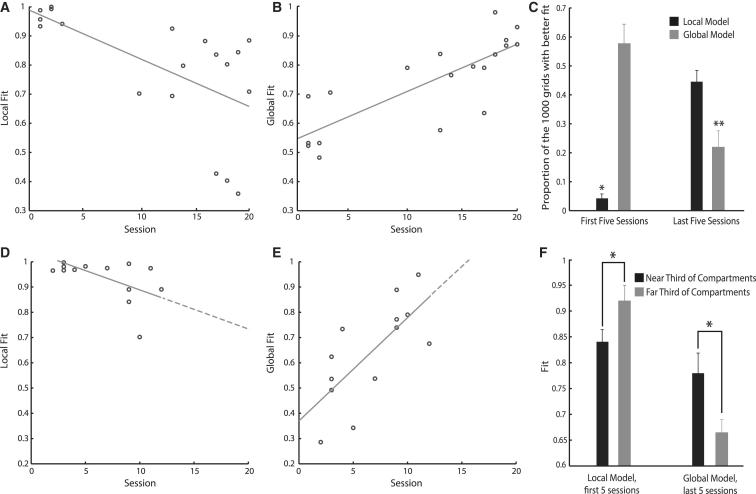
The Transition from Local to Global Representations Cannot Be Explained by Biases in the Sampling of Grid Cells (A and B) The fit between recorded firing patterns of grid cells of a single scale and ideal local and global grids, respectively, as a function of experience of the environment. Only cells with a scale of 45 to 55 cm in the screening environment are included. Each data point represents the average local and global fits across all 45–55 cm cells with an independent fit >0.45, recorded from one animal in one session. (C) The proportion of 1,000 ideal grids, with random phase offsets between the compartments, with a better fit to the cells in (A) and (B) than the local or global models. Values are mean + SEM across 45–55 cm cells with an independent fit >0.45 in the first or last five sessions. WSRTs compare observed values to an expected median of 0.5. (D and E) The fit between recorded firing patterns of grid cells from a single module in a single animal and ideal local and global grids, respectively, as a function of experience. Dashed lines extend the least-squares lines to predict local and global fits in unrecorded sessions. (F) The best fit achieved by the local model in the first five sessions and the global model in the last five sessions to the grid patterns in the thirds of the compartments nearest to or furthest from the corridor. Values are mean + SEM of the collapsed average within animals of cells with an independent fit >0.45. Paired, one-tailed t tests test whether difference in observed means differs from an expected mean of 0. ^∗^p < 0.05; ^∗∗^p < 0.01.

Grid cells are organized into functionally distinct modules [9, 18]. These modules are distributed non-uniformly in the brain, with modules in dorsal areas of mEC often having smaller-scale firing patterns than those found more ventrally [1, 18]. Here, as is typical of recordings in mEC, electrodes were implanted dorsally and advanced ventrally to locate grid cells. As such, the transition from a local to a global representation could be explained by biases in the sampling of modules across time. Specifically, if smaller-scale grid modules formed local representations and larger-scale modules formed global representations, the dorsal-to-ventral sampling bias could produce an artifactual local-to-global representation shift. To eliminate this possibility, we repeated the prior analysis separately for cells with scale either above or below the median grid scale. Both groups exhibited a significant shift from local to global representations (Figures S2E and S2F). However, as we did not record any grid cells with a scale less than the median in the last five sessions, we further analyzed the single grid scale (45–55 cm) for which grid cells with an independent fit >0.45 were recorded throughout the experiment. Again, the local model’s fit decreased with experience (r = −0.6185, r^2^ = 0.3826, p = 0.0048; Figure 3A), while the global model’s fit increased (r = 0.8001, r^2^ = 0.6402, p = 3.902 × 10^−5^; Figure 3B). Indeed, within this scale, grid firing patterns changed with experience from significantly more local to significantly more global than expected by chance between the first and last five sessions (Figure 3C). The same transition was also evident when analysis was restricted further to grid cells from a single module within an individual animal. In the animal with the most sequential recordings of grid cells from a single grid module and with an independent fit >0.45 (ten sessions), a two-way ANOVA revealed an interaction between experience of the environment and the goodness of fit of the two models (session × model, F_(8,25)_ = 9.99, p = 0.0019; Figures 3D and 3E), with the global model’s fit increasing significantly with experience (r = 0.7188, r^2^ = 0.5167, p = 0.0056; Figure 3E). That the transition from local to global representations is apparent within individual grid scales and modules demonstrates that it cannot be explained simply by biases in the sampling of grid scales and modules across time.

Grid cell firing likely derives from path integration: utilizing information about self-motion to update a representation of self-location [5, 19–22]. We therefore asked whether there was any difference in the grid representations between the thirds of each compartment closest to and furthest from the corridor. We hypothesized that the reduced distance between the sections of the compartments nearest the corridor may result in the accumulation of less path integration error and so produce more accurate global representations than in the sections furthest away. Confirming this hypothesis, in the first five sessions, grid patterns in the near third of each compartment were significantly less local than those in the furthest third (paired one-tailed t test, t_(17)_ = −1.931, p = 0.0352; Figure 3F), while in the last five sessions, grid patterns in the thirds closest to the corridor were significantly more global than those in the furthest thirds (paired one-tailed t test, t_(10)_ = 1.959, p = 0.0392; Figure 3F).

## Discussion

Grid cells are of great interest to computational neuroscientists as their periodicity allows them to form highly efficient spatial representations and to act as a metric for spatial calculations [5–7]. However, such calculations would be prone to significant errors where grid firing diverges from a regular and continuous pattern due to distortions and discontinuities. During initial exposures to the multicompartment environment, grid patterns were dominated by local sensory cues, replicating between the two compartments. However, with increasing experience, discontinuities in grid cell firing patterns between the compartments were incrementally reduced to form a single, continuous representation that spanned both compartments. This transition suggests grid cells adjust their firing to produce the globally coherent representation required for them to act as an effective spatial metric. Though the mechanism underpinning this transition remains undetermined, for grid cells to form a coherent global representation, it is necessary for them to identify the relative positions of all points in the environment. Models of grid cell formation largely describe their firing in terms of self-motion [5, 19–22], and the integration of self-motion as the animal explores the environment (path integration) is one way in which the relative position of points in space can be discerned. The more globally coherent firing patterns observed in the sections of the compartments closest together gives credence to this explanation: if the global coherence of grid patterns did not depend on path integration, one would not expect any difference across the compartments. The highly extended time frame over which cells transitioned from a local to a global representation made the continual recording from single cells across the whole period difficult. However, single grid modules, which are thought to form functional units [4, 9, 18], appeared to adjust their representations gradually and continuously (Figures 3D and 3E). Further, the firing fields of one grid cell recorded across 15 consecutive sessions appeared to shift continuously, rather than undergo a sudden transformation (Movie S1). These observations argue against an abrupt change in representation. However, to fully understand the temporal dynamics of the change from a local to a global representation, one would have to follow the same population of cells throughout the transition. The grid cells’ gradual disambiguation of the compartments across sessions was reminiscent of the slow transition of place fields in morphed environments from boundary-referenced replication to direct remapping [23, 24], a process likely indicative of a slow plasticity-based mechanism [24, 25]. The common slow transition among grid and place cells likely indicates a unifying underlying mechanism, though it remains to be seen whether changes in grid cell firing preempts or indeed drives changes in place cell firing.

## Experimental Procedures

For the full experimental protocol, please see Supplemental Experimental Procedures.

### Animals and Surgery

All work was carried out under the Animals (Scientific Procedures) Act 1986 and according to Home Office and institutional guidelines. One or two microdrives were implanted above mEC in eight male Lister-Hooded rats.

### Electrophysiological Recording and Experimental Protocol

Each session began with a 20-min baseline trial in which electrophysiological and positional data were acquired while animals foraged in a 1 × 1 m environment. Following identification of grid cells, rats were recorded while foraging in the multicompartment environment. Comprising two 90 × 90 × 50 cm compartments connected by a 180 × 40 × 50 cm corridor, the multicompartment environment was designed such that the two adjacent compartments would be as perceptually identical as possible.

Each recording session consisted of two 40-min trials, with the compartments’ positions switched and the floor of the environment rotated between trials to control unidentified sensory cues distinguishing the compartments. Animals ran at most one session per day for a maximum of 20 sessions.

### Analyses

#### Spike Sorting, Binning, and Grid Cell Inclusion Criteria

Spike sorting was performed offline using the automated clustering algorithm KlustaKwik [26].

Two-dimensional firing rate maps were calculated by assigning recorded positions and spikes to 2 × 2 cm bins covering the environment and dividing the number of spikes in each bin by the cumulative dwell time in each bin.

For inclusion in subsequent analysis, putative grid cells were first assessed using a gridness measure [27]. Cells were considered grid cells if their gridness exceeded the 99^th^ percentile of a shuffled distribution of 1,000 gridness scores calculated from rate maps where spike times were randomly offset relative to position by at least 20 s. 85 cells passed these criteria, with a mean gridness of 0.89 in the baseline environment.

#### Correlations

To measure the similarity of grid firing between the two compartments, we calculated a Pearson product-moment correlation coefficient comparing firing rates in equivalent bins in the rate maps of the two compartments.

#### Fitting of Ideal Grids

Recorded rate maps were fitted by ideal grid patterns to determine whether grid representations were referenced to local or global features of the environment. In the local model, recorded rate maps were fitted by ideal grids whose firing patterns replicated between the two compartments. In contrast, ideal grids fitted in the global model consisted of a single grid pattern spanning both compartments. The less constrained independent model allowed identification of the best possible fit between each recorded rate map and any ideal grid. The independent model was therefore used to discard cells too irregular to be well fit by any model and to normalize fits in the local and global models.

## Author Contributions

F.C. collected the data. F.C., N.B., and C.B. analyzed the data and wrote the paper. D.M. contributed to data collection. F.C., K.J., N.B., and C.B. conceived and designed the experiment.

## Supplemental Information

Document S1. Supplemental Experimental Procedures and Figures S1–S3

Movie S1. The Firing Fields of a Single Grid Cell Recorded across 15 Sessions Appear to Shift Gradually and Continuously Rather Than Abruptly, Related to Discussion Here, in the right-hand panel, the firing rate maps of a single grid cell recorded across 15 sessions are shown sequentially. Each rate map is the recorded firing of the cell in the second trial of each session. In the left-hand panel, the spatial correlation of firing rates in equivalent bins of the two compartments is plotted as a function of experience of the environment. Each data point is the spatial correlation for that trial alone.

Document S2. Article plus Supplemental Information

## References

[1] Hafting T., Fyhn M., Molden S., Moser M.-B., Moser E.I. (2005). Microstructure of a spatial map in the entorhinal cortex. Nature.

[2] Boccara C.N., Sargolini F., Thoresen V.H., Solstad T., Witter M.P., Moser E.I., Moser M.-B. (2010). Grid cells in pre- and parasubiculum. Nat. Neurosci..

[3] Fyhn M., Hafting T., Treves A., Moser M.-B., Moser E.I. (2007). Hippocampal remapping and grid realignment in entorhinal cortex. Nature.

[4] Yoon K., Buice M.A., Barry C., Hayman R., Burgess N., Fiete I.R. (2013). Specific evidence of low-dimensional continuous attractor dynamics in grid cells. Nat. Neurosci..

[5] McNaughton B.L., Battaglia F.P., Jensen O., Moser E.I., Moser M.-B. (2006). Path integration and the neural basis of the ‘cognitive map’. Nat. Rev. Neurosci..

[6] Buzsáki G., Moser E.I. (2013). Memory, navigation and theta rhythm in the hippocampal-entorhinal system. Nat. Neurosci..

[7] Fiete I.R., Burak Y., Brookings T. (2008). What grid cells convey about rat location. J. Neurosci..

[8] O’Keefe J., Nadel L. (2008). The Hippocampus as a Cognitive Map.

[9] Barry C., Hayman R., Burgess N., Jeffery K.J. (2007). Experience-dependent rescaling of entorhinal grids. Nat. Neurosci..

[10] Stensola T., Stensola H., Moser M.B., Moser E.I. (2015). Shearing-induced asymmetry in entorhinal grid cells. Nature.

[11] Krupic J., Bauza M., Burton S., Barry C., O’Keefe J. (2015). Grid cell symmetry is shaped by environmental geometry. Nature.

[12] Derdikman D., Whitlock J.R., Tsao A., Fyhn M., Hafting T., Moser M.B., Moser E.I. (2009). Fragmentation of grid cell maps in a multicompartment environment. Nat. Neurosci..

[13] Skaggs W.E., McNaughton B.L. (1998). Spatial firing properties of hippocampal CA1 populations in an environment containing two visually identical regions. J. Neurosci..

[14] Lever C., Burgess N., Cacucci F., Hartley T., O’Keefe J. (2002). What can the hippocampal representation of environmental geometry tell us about Hebbian learning?. Biol. Cybern..

[15] Spiers H.J., Hayman R.M.A., Jovalekic A., Marozzi E., Jeffery K.J. (2015). Place field repetition and purely local remapping in a multicompartment environment. Cereb. Cortex.

[16] Hafting T., Fyhn M., Bonnevie T., Moser M.-B., Moser E.I. (2008). Hippocampus-independent phase precession in entorhinal grid cells. Nature.

[17] Domnisoru C., Kinkhabwala A.A., Tank D.W. (2013). Membrane potential dynamics of grid cells. Nature.

[18] Stensola H., Stensola T., Solstad T., Frøland K., Moser M.-B., Moser E.I. (2012). The entorhinal grid map is discretized. Nature.

[19] O’Keefe J., Burgess N. (2005). Dual phase and rate coding in hippocampal place cells: theoretical significance and relationship to entorhinal grid cells. Hippocampus.

[20] Fuhs M.C., Touretzky D.S. (2006). A spin glass model of path integration in rat medial entorhinal cortex. J. Neurosci..

[21] Burak Y., Fiete I.R. (2009). Accurate path integration in continuous attractor network models of grid cells. PLoS Comput. Biol..

[22] Etienne A.S., Jeffery K.J. (2004). Path integration in mammals. Hippocampus.

[23] O’Keefe J., Burgess N. (1996). Geometric determinants of the place fields of hippocampal neurons. Nature.

[24] Lever C., Wills T., Cacucci F., Burgess N., O’Keefe J. (2002). Long-term plasticity in hippocampal place-cell representation of environmental geometry. Nature.

[25] Barry C., Burgess N. (2007). Learning in a geometric model of place cell firing. Hippocampus.

[26] Kadir S.N., Goodman D.F.M., Harris K.D. (2014). High-dimensional cluster analysis with the masked EM algorithm. Neural Comput..

[27] Sargolini F., Fyhn M., Hafting T., McNaughton B.L., Witter M.P., Moser M.-B., Moser E.I. (2006). Conjunctive representation of position, direction, and velocity in entorhinal cortex. Science.

