# Supplementary material for: Grid Cells Form a Global Representation of Connected Environments
Source: Curr Biol. 2015 May 4;25(9):1176–82. doi: 10.1016/j.cub.2015.02.037 (PMC4425461; doi:10.1016/j.cub.2015.02.037)
Supplement: Document S1. Supplemental Experimental Procedures and Figures S1–S3 [file mmc1.pdf]

Current Biology

Supplemental Information

Grid Cells Form a Global Representation of Connected Environments

Francis Carpenter, Daniel Manson, Kate Jeffery, Neil Burgess, and Caswell Barry

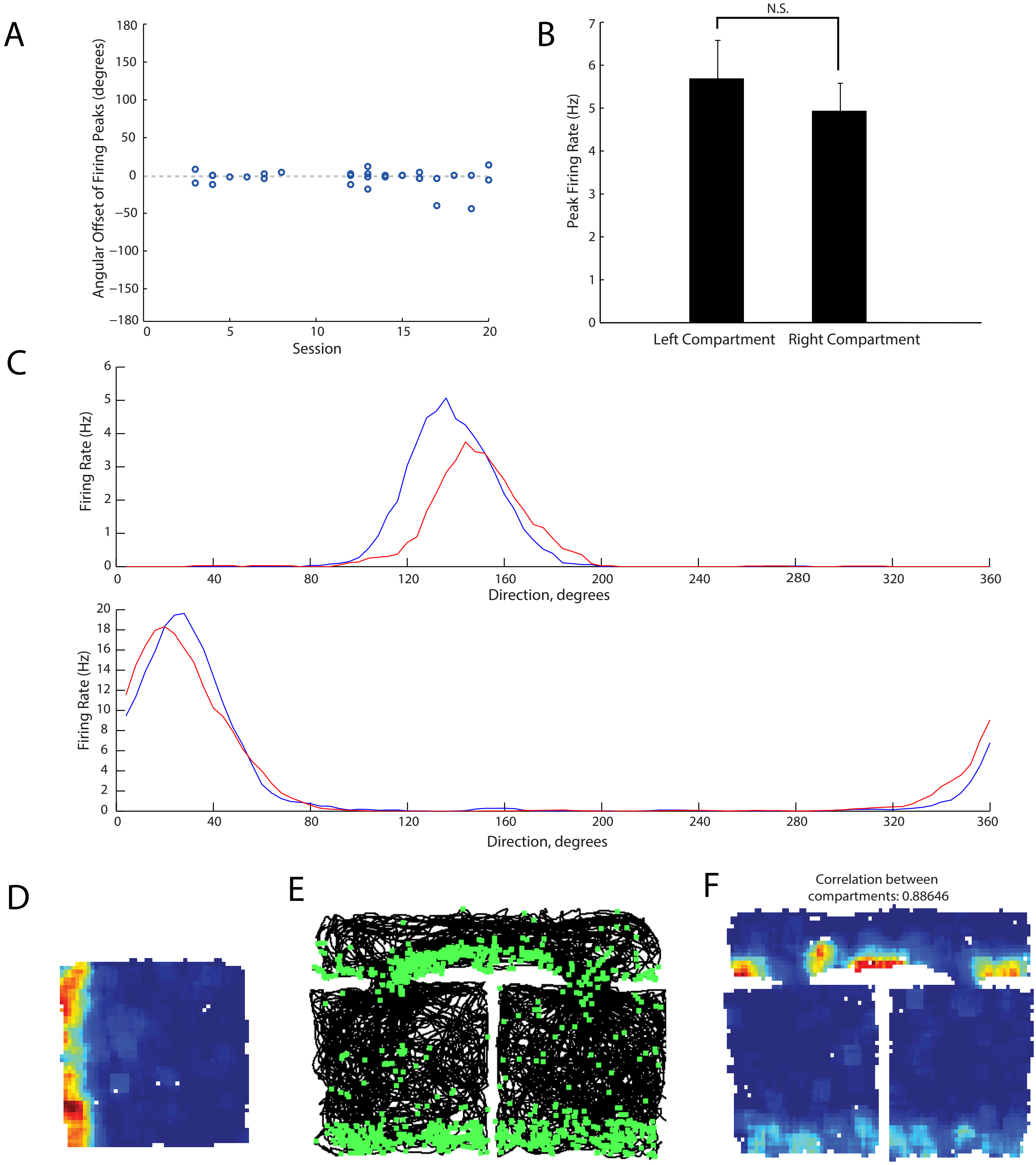

Supplementary Figure 1: Head direction and border cell firing replicates in the two compartments regardless of experience: Further data relevant to Figure 1.

A, B and **C**: Head direction cell firing replicated in the two compartments regardless of experience. **A**. The angular offset between the firing rate peaks of directional ratemaps from the left and right compartments. Angular offsets were calculated by producing a directional cross-correlogram between directional firing rate maps of the two compartments and calculating the distance from the origin to the cross-correlogram peak. For each recorded head direction cell offsets are plotted as a function of experience of the environment. A negative angular offset indicates the firing peak in the left compartment is anticlockwise of the firing peak in the right compartment. Peak offsets are clustered around 0º regardless of experience, and the correlation between peak offset and session was not significant (r = -0.1744, p = 0.3481). **B**. Peak directional firing rates were also consistent between the compartments. Plotted values are mean + sem (Wilcoxon signed rank test, z = 1.666, p = 0.0958). **C**. Example directional firing rate maps for two representative head direction cells recorded from two rats. The directional firing rate map of the left compartment is plotted in blue and the right compartment in red. **D, E** and **F**: A putative border cell recorded in session 11 showed replicated firing between the two compartments of the multicompartment environment. **D**. The firing rate map of a putative border cell recorded in the screening environment. Higher firing rates are indicated by hotter colours, unvisited bins are white. **E**. The unbinned path of the animal as it explores the multicompartment environment in black, overlaid in green with the location of spikes from the same cell as in **D**. **F**. Firing rate map of the same putative border cell. The cell was recorded in the 11th session, yet like head direction cells, even with extended experience, shows highly consistent firing in the two compartments.

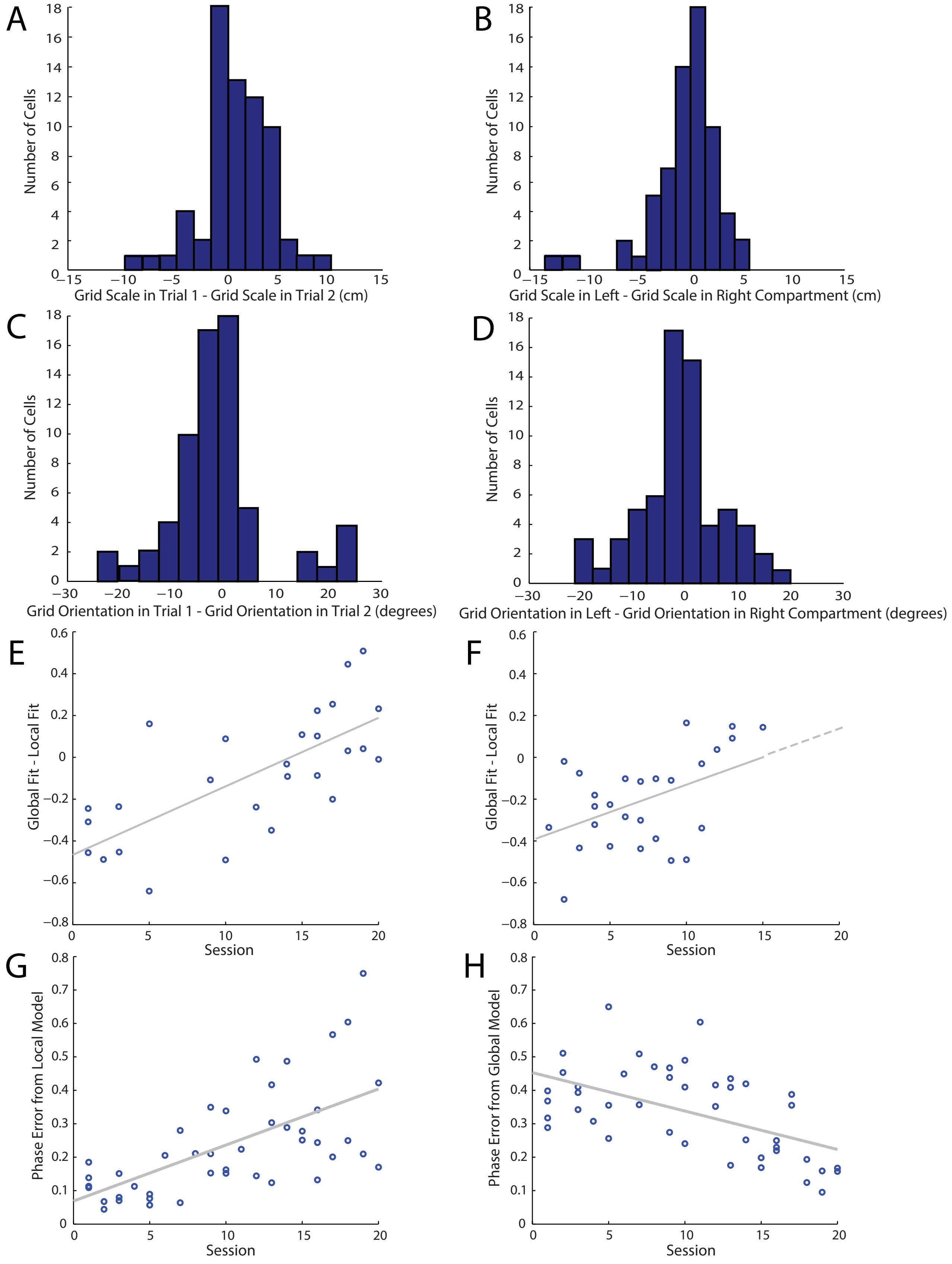

Supplementary Figure 2: Grid properties are consistent in the two compartments. Analysis of grid phase confirms local to global representation transition: Further data relevant to Figure 2.

A - D: Grid properties are consistent in the two compartments. For each grid cell recorded in both trials of a single session, the differences in grid scale (**A**, **B**) and orientation (**C**, **D**) between trial 1 and trial 2 and between the left and right compartments were calculated from each trial's spatial autocorrelogram. Only cells with a fit of the independent model of 0.45 were included to discard cells which were sufficiently irregular that estimates of grid properties were inaccurate. To analyse whether the difference in grid scale and orientation between compartments was greater than that between trials, Wilcoxon signed-rank tests compared the medians of the absolute (the magnitude of the) differences between trials and between compartments. The differences in grid scale between compartments and between trials were not significantly different (z = -0.7397, p = 0.4597). Neither did the differences in grid orientation differ significantly (z = 0.8982, p = 0.3691). **E** and **F**: both small and large scale grid cells transition from local towards global representations. In **E** and **F** the difference in fits between the global and local model (Global - Local) is plotted as a function of experience of the environment separately for grid cells with scale greater than the median grid scale (**E**) and for grid cells smaller than the median grid scale (**F**). A significant positive correlation is observed in both larger (r = 0.6862, r^2 = 0.4709, p = 0.0001) and smaller (r = 0.5057, r^2 = 0.2557, p = 0.0071) scale grid cells. Data points represent the average fit across all cells recorded in one animal in one session. **G** and **H**: Phase offset analysis confirms transition from a local to a global representation. In **G** and **H**, phase error equates to the magnitude of the vector in grid-phase space connecting the observed phase in the right-hand compartment to the phase predicted under local or global models based on grid parameters estimated from firing fields in the left-hand compartment (see Supplementary Methods). Each data point represents the average phase error for all cells recorded from one animal in one session. In **G**, the phase error from the local model increases significantly as a function of experience of the environment (r = 0.6274, r^2 = 0.3936, p = 5.179x10^{-7}), indicating the grids become less local with experience. In contrast, in **H** the phase error from the global model decreases across sessions (r = -0.5514, r^2 = 0.3040, p = 1.048x10^{-4}), confirming that grids become more global with experience.

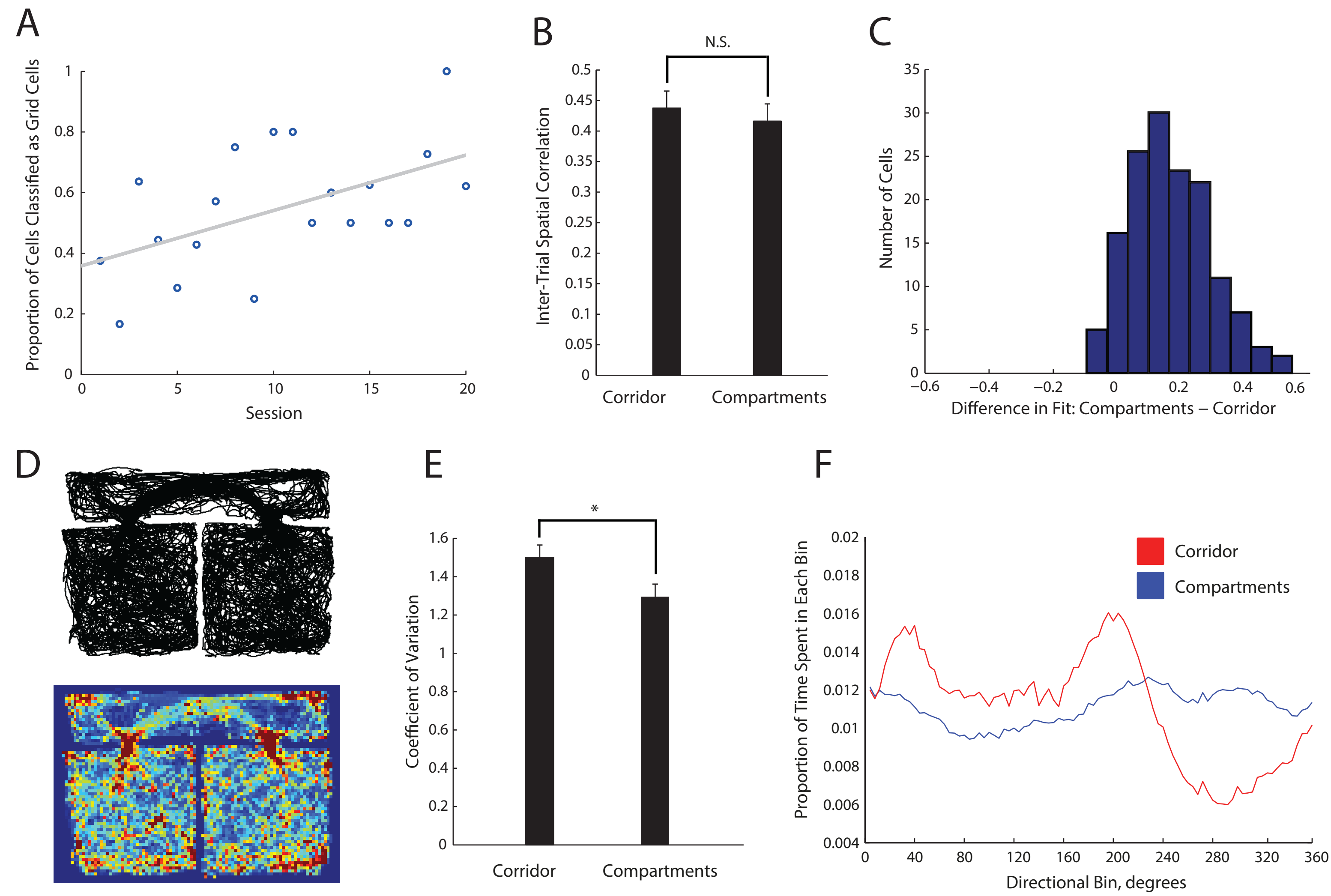

Supplementary Figure 3: Firing patterns in the corridor are 'grid-like' and stable, but irregular, co-occurring with highly stereotyped behaviour: Further data relevant to Figure 2.

A. The proportion of cells designated as grid cells by a Grid Cell Classifier analysing firing in the corridor (see Supplementary Methods) indicates the presence of grid-like firing in the corridor, which becomes more pronounced with experience of the environment (r = 5238, r^2 = 0.2744, p = 0.0177). **B**. The spatial-correlation of firing rates in trial 1 with firing rates in equivalent positions in space in trial 2, calculated separately for the compartments and corridor. Data are presented as mean + sem across all cells recorded in both trials of a single session. Firing was equally stable in the compartments and corridor (*paired t-test*, t_{114} = 1.278, p = 0.2037). **C**. The best fit achieved between ideal grids and recorded firing patterns in the corridor or compartments. Ideal grids fitted were required to have the same orientation and scale as was previously found to achieve the best independent fit. The difference in best fit between the corridor and compartments for all grid cells is plotted in a histogram. The best observed fit to firing in the corridor was significantly lower than that to the compartments (*unpaired t-test*, t_{143} = 14.52, p = 6.055x10^{-30}). **D**. Top: the path taken by an animal in a typical, 40 minute recording trial. Bottom: Heat map of unsmoothed dwell times for the same trial, hotter colours indicate longer dwell. **E**. The coefficient of variation ($C_v = \sigma / \mu$) of dwell time between bins, averaged across all recording trials. The coefficient of variation was significantly greater in the corridor than the compartments (*paired t-test*, t_{85} = 3.7328, p = 3.414x10^{-4}), indicating greater variability in dwell times between bins in the corridor than compartments. **F**. The mean proportion of time spent in each head direction bin across all animals in the corridor (red) and compartments (blue) differed significantly between the corridor and the compartments (*Kolmogorov-Smirnov test*, p = 4.352x10^{-4}). In the corridor large biases in head direction are evident, likely due to the ballistic running between the compartments evident in **D**. In the compartments behaviour is less stereotyped, and the animals sampled all spatial and directional bins more evenly.

Supplementary Methods:

Animals

All work was carried out within the terms of the Animals (Scientific Procedures) Act 1986, and according to Home Office and institutional guidelines. 8 experimentally naïve, male Lister Hooded rats (275-400g at surgery) were housed communally under a 12:12 inversed light-dark cycle for at least one week prior to surgery. Following surgery, animals were held individually in Perspex cages and restricted to 90% of their free-feeding body weight.

Microdrives and surgery

During surgery, rats received either 1 or 2 custom-made microdrives. Each microdrive consisted of 32 HML-coated 17-μm Platinum-Iridium (90-10%) electrodes twisted into 8 separate tetrodes. The electrodes were mounted on a single drive-mechanism (tetrodes not independently adjustable) which allowed for their advancement through the turning of a trapped screw. Before surgery electrode tips were electroplated in a Platinum solution to <150kΩ impedance.

Microdrives were implanted following the same surgical procedures as described previously [S1]. In four rats, a trephine was used to drill a single craniotomy above dorso-lateral medial entorhinal cortex (mEC). In two rats, craniotomies were drilled bilaterally above dorso-lateral mEC in both hemispheres. In the final two rats, craniotomies were drilled above mEC in one hemisphere and the Hippocampus (HPC) in the other. For mEC implants, electrodes were angled anteriorly 8-10° and implanted 4.5mm lateral to Bregma, 0.3mm anterior to the transverse sinus and at a depth of 1.6mm from the surface of the brain. Following surgery animals were allowed at least a one week recovery period in which Baytril (enrofloxacin – 4ml/100ml water) was given in the water to prevent infection and Metacam (meloxicam – 5mg/kg) suspended in jelly acted as an analgesic.

Electrophysiological recording and behavioural training

Electrophysiological and positional data was acquired using the DACQ (Axona Ltd., St Albans, UK) recording system, details of which are described in Barry et al. [S1].

Neural activity was recorded while animals were trained to forage for sweetened rice in a square 1mx1m environment comprising four 60cm high grey walls held together by corner clamps. The environment was placed on an earthed black metal-composite sheet in the centre of a room containing a number of distal landmarks to aid orientation. Both the walls and floor were cleaned between recording trials. Electrodes were advanced in 30-60µm steps between recordings until grid cells were identified.

Experimental environment and protocol

Each recording session began with a 20 minute baseline trial in the square screening environment. Following identification of grid cells, rats were placed in an enclosed white-plastic travel box while the experimental environment was prepared. Comprising two 90x90x50cm compartments connected by a 180x40x50cm corridor, the multicompartment environment was designed such that the two adjacent compartments would be as perceptually identical as possible. Painted matte black, the environment was placed on a transparent-Perspex floor on top of a uniform black plastic groundsheet, and surrounded by four black curtains organised in a square. Each compartment contained a light at the centre of the south wall, 40cm from the floor. Each light was a battery-powered bike light mounted to the outside of the compartment and enclosed in aluminium-foil. The light entered each compartment solely through a 3cm diameter window covered with translucent frosted-Perspex. No other light source was present. Into the north face of each compartment was cut a trapezoidal doorway, measuring 10cm across at its base and 50cm across at its top. Each compartment was reinforced along its base with a 1cm strip of stainless steel, allowing them to be easily moved without changing shape.

At the beginning of each experimental session the floor of the environment was cleaned. The first experimental trial was then started, with the rat being placed in the corridor between the two compartments, facing the north wall. Throughout the trial the experimenter moved pseudo randomly around the circumference of the environment, distributing rice such that the rat explored the whole arena. The tether of the recording system was counterbalanced and suspended above the animal using 'runners' such that the centre of mass of the tether moved freely as the animal did, ensuring no directional cue was provided. After 40 minutes, or when the rat had satisfactorily covered the entire environment, the rat was returned to its travel box. The compartments were then removed and the Perspex floor was rotated 180° before being cleaned. Once dry, the compartments were replaced in the opposite positions to the first trial, such that the compartment in the west of the environment during the first trial was now in the east and vice versa. The animal was then again placed in the corridor facing the north wall and the second experimental trial began. Rats ran at most one session per day for a maximum of 20 sessions. Recordings in the multicompartment environment continued on subsequent days as long as at least one grid cell was still identifiable during screening. If no grid cell was present, the electrodes were again moved until further grid cells were found.

Spike sorting and binning

Spike sorting was performed offline through fitting a mixture of Gaussians according to an expectation-maximisation algorithm using KlustaKwik [S2]. Putative clusters were then further analysed using the data analysis suite Tint (Axona Ltd., St Albans, UK). In particular, Tint was used to coordinate clusters across trials and to correct for over-clustering according to the amplitude, waveform and temporal autocorrelation of spikes.

For two-dimensional firing rate maps the animals' recorded positions and spikes were assigned to 2x2cm bins covering the environment. Unsmoothed firing rate maps were calculated by dividing the number of spikes assigned to each bin by the cumulative dwell time in each bin. Smoothed ratemaps

were constructed using a 5x5bin boxcar filter, with the firing rate in bin i equal to the number of spikes in the kernel centred on i divided by the occupancy of the kernel.

For directional ratemaps, recorded heading directions and spikes were assigned to 4° bins between 0 and 360°, with the animal's head direction calculated from the angular offset of two groups of LEDs on the animal's head-stage. Unsmoothed ratemaps were constructed by dividing the number of spikes by the cumulative dwell in each directional bin. The Kullback-Leibler Divergence (KL Divergence) was used to measure the divergence between the resulting circular ratemap and a uniform circular distribution. Cells were classified as head direction cells where the KL Divergence in the screening environment exceeded 0.25.

Spatial Autocorrelograms and Grid Cell Inclusion Criteria

For inclusion in subsequent analysis, putative grid cells were assessed using a gridness measure, following Sargolini et al. 2006 [S3]. For both the screening environment, and for each compartment of the multicompartment environment, a spatial autocorrelogram was constructed from the smoothed ratemap, according to:

$$r(\tau_x,\tau_y) = \frac{n\Sigma\lambda(x,y)\lambda(x-\tau_x,y-\tau_y) - \Sigma\lambda(\mathrm{x,y})\Sigma\lambda(\mathrm{x}-\tau_\mathrm{x},y-\tau_y)}{\sqrt{n\Sigma\lambda(x,y)^2 - \left(\Sigma\lambda(x,y)\right)^2}\,.\sqrt{n\Sigma\lambda\left(x-\tau_x,y-\tau_y\right)^2 - \left(\Sigma\lambda\left(x-\tau_x,y-\tau_y\right)\right)^2}}$$

Where $r(\tau_x,\tau_y)$ is the autocorrelation between bins with spatial offset τ_x and τ_y. $\lambda(x,y)$ is the firing rate in bin (x,y), while n is the total number of bins. The six local maxima closest to but excluding the origin of the autocorrelogram were used to identify the orientation, scale and gridness of the putative grid cell. Orientation was measured as the angle from a consistent arbitrary horizontal reference line to the first peak of the autocorrelogram in an anticlockwise direction. The median distance from the origin to the six peaks was used to estimate the scale of the putative cell. Finally, the gridness was calculated by rotating the autocorrelogram in 30°steps for 150° and taking

the Pearson product-moment correlation coefficient between each rotated autocorrelogram and the un-rotated autocorrelogram. The maximal correlation obtained at 30, 90 or 150° was subtracted from the minimum correlation found at 60 or 120° to produce the gridness score. When analysing the gridness of firing patterns in the multicompartment environment, gridness scores were calculated separately for each compartment and subsequently mean averaged.

For each putative grid cell the gridness score obtained from the screening ratemap or the multicompartment ratemap was compared to the distribution of gridness scores observed following 1000 shuffles of the positional and spiking data of the same cell during the same trial. That is, for each shuffle the relative timing of the position and spike data was circularly shifted randomly by at least 20 seconds, with the autocorrelogram and gridness then calculated using the ratemap resulting from each shift. To be considered a grid cell and included in subsequent analysis, a cell was required to have a gridness score in the 99th percentile of the distribution of shuffled gridness scores in either the screening or multicompartment environment.

To ensure that no single grid cell recorded across tetrodes was considered two separate cells, where any putative cells with similar firing fields were observed within or between tetrodes a spatial cross-correlation was first performed. If the two putative grid cells had consistent spatial firing the temporal cross-correlation between the cells was subsequently analysed. Where pairs of putative grid cells had both high spatial and temporal cross-correlations they were considered to be single cells.

85 grid cells passed these inclusion criteria, with a mean average gridness of 0.89 in the screening environment.

Analyses

i. General

The 85 grid cells were recorded across 99 sessions. There were 59 instances of recording a cell in a session in which that cell had been recorded in at least one previous session. In total, there were 144 grid cell/session conjunctions (85 unique cells + 59 instances of re-recording). In 115/144 of the cell/session conjunctions the grid cell was held across both multicompartment trials. Here, any calculated metric is mean-averaged across the two trials, yielding a single value for every cell in each session.

Unless specifically analysing the corridor, all other analyses discarded the corridor and analysed only ratemaps formed by the left and right compartments separated by the gap created by the central wall. While firing in the corridor was grid-like - formed of peaks and troughs in firing (Supplementary Fig 3A), and stable (Supplementary Fig 3B), firing in the corridor was significantly less regular and hexagonal than the compartments (Supplementary Fig 3C). This irregularity is consistent with past recordings of grid cells in linear environments [S4, S5], and was likely exacerbated by highly stereotyped behaviour in the corridor: In the corridor animals tended to run in a ballistic fashion between the compartments, resulting in a highly uneven sampling of space and direction (Supplementary Fig 3D-F). As any ideal grid could only be poorly fitted to the corridor, the comparison of alternative models through the fitting of ideal grids was uninformative.

ii. Correlations

To measure the similarity of grid cell representation between the left and right compartments a Pearson product-moment correlation coefficient was calculated comparing the firing rates in equivalent bins of the smoothed ratemaps of the two compartments. Bins were discarded if they were unvisited in either compartment or they had a firing rate of 0 in both compartments.

Further correlations were used to assess whether grid cell representations tracked the movement of the compartments between trials. For each cell recorded across both trials in a single day Pearson product-moment correlation coefficients were calculated between firing rates in equivalent bins in the same compartment in successive trials (compartment-wise correlation), or between equivalent bins in the same position in global space in successive trials (space-wise correlation).

iii. Fitting of ideal grids

Ideal grid patterns were fitted to ratemaps according to three models to analyse whether the recorded grid representations could be best described as being determined by local or global features of the environment. The recorded grid firing patterns were first fit by the 'independent' model. Here, 900 ideal grids were generated, each the product of three cosine gratings with wave vectors k_1, k_2 and k_3 and phase offsets c_1, c_2 and c_3. Wave vector $\vec{k} = \left(\frac{2\pi}{\lambda}\cos(\varphi), \frac{2\pi}{\lambda}\sin(\varphi)\right)$ where λ is the grating wavelength ($\lambda = G\sqrt{3}/2$, where G is the grid scale), and φ is the grating orientation. The three wave vectors form a regular triangular grid where orientations differ by $\frac{2\pi}{3}$ (i.e., 120°) and $c_1 + c_2 = c_3$. As such, firing rate as a function of location is given by:

$$f(\vec{x}) = A\left(1 + \cos\left(\overrightarrow{k_1}.\vec{x} + c_1\right)\right)\left(1 + \cos\left(\overrightarrow{k_2}.\vec{x} + c_2\right)\right)\left(1 + \cos\left(\overrightarrow{k_3}.\vec{x} + c_3\right)\right)$$

where A determines the peak firing rate ($8A$) and does not affect spatial correlations with $f(\vec{x})$. Each of the 900 ideal grids was a unique combination of one of 30 increments of scale and 30 increments of orientation. Orientation varied in increments of 2° between 0 and 60°, while scale varied in equal increments between the scale of the cell as estimated from the screening autocorrelogram ±20%. Spatial cross-correlograms between the ideal grid and the recorded ratemap were used to find the Pearson product-moment correlation at all spatial offsets, ignoring unvisited bins. From the spatial

cross-correlogram the phase offsets which yielded the highest correlation for that orientation and scale combination were then identified. The independent fit value was then calculated as the highest correlation found between the independent model and the ratemap across all combinations of scale, orientation and phase. The independent model was used to assess the scale and orientation of the recorded grid, with subsequently fitted 'local' and 'global' models constrained to the orientation and scale achieving the best fit in the independent model. Further, as the independent fit indicates the highest correlation that can be achieved between any ideal grid and the ratemap, grid cells with an independent fit <0.45 were excluded from subsequent analysis to account for cells which could not be well fitted by any model. A criterion of 0.45 was chosen as it was near the median independent fit of 0.4702. As such, just under half of grid cell/session conjunctions were excluded (65/144, 45.14%). Varying the required inclusion criterion between 0.4 and 0.5 had little effect on the overall conclusions drawn from the results.

To subsequently fit the local and global models two additional ideal grids were generated, each with the same orientation and scale as was found to achieve the best independent fit. In the local model the ideal grid had the same phase in the two compartments, whereas the global grid had a continuous phase across both compartments. The Pearson product-moment correlations between the firing rate map and the local and global ideal grids was then calculated at each offset as above. The maximum correlations achieved between the local and global grid and the recorded firing pattern were then identified. These maximum correlations were divided by the maximum correlation found under the independent fit, giving the local and global fit values, with which comparisons could be made across cells.

To assess the significance of the local and global fits, each recorded ratemap was also fitted with 1000 randomly phase offset grids. As in the local and global models each of the randomly offset grids was constrained to the same orientation and scale as was found under the independent model. However, each had a random phase offset (0-2π along the first two grid axes) between the two

compartments. For each of the 1000 grids a spatial cross-correlogram with the recorded ratemap was calculated to find the spatial offset which achieved the maximum correlation between that ideal grid and the ratemap. Again, as in the local and global model, the maximum correlation at any offset was divided by the independent fit to yield the fit value for each randomly offset grid. For each cell/session conjunction we then calculated the proportion of the 1000 randomly phase offset grids which achieved a fit greater than that of the local or global model. That is, we identified where the local and global fits fell in the distribution of fits achieved by the 1000 randomly offset grids. These values were then collapsed (mean averaged) within animals to create a single value for each animal in every session in which grid cells were recorded. Under the null hypothesis of no particular phase relationship between the grid firing in each compartment, a Wilcoxon signed rank test was used to examine whether the observed proportions of the 1000 grids with a better fit than the local or global model differed significantly from 0.5. This test was applied in the first and last five sessions.

To identify whether the apparent transition between local and global representations could be explained by biases in the sampling of different grid modules across time we repeated the above analyses on particular subsets of the data. First, the analysis was repeated including only those cells with a scale of 45-55cm, as estimated from the spatial autocorrelogram calculated from the screening environment's ratemap. In addition, the analysis was repeated on only those grid cells from a single animal in which a number of cells with independent fits > 0.45 and from a single module were recorded over a number of sessions. Grid cells were assigned to putative modules based on the scale of the cell in the screening environment. Concurrently recorded cells were considered to be of the same grid module if the ratio of the larger to the smaller scale cell was less than 1.4 [S6].

Finally, we fitted recorded grids in the same way as described above, except the thirds of each compartment closest to and furthest from the corridor were fitted separately. Again, for each recording we discarded the corridor to produce a ratemap comprising both compartments separated

by the gap due to the dividing walls. Here, we then divided the two compartments into three sections according to distance from the corridor to produce three ratemaps of ~180x30cm. Idealised grids were then fitted to the thirds closest to and furthest from the corridor for each cell under the same principles as above. Again, fits were collapsed within animals such that each data point corresponded to the average of all cells recorded from one animal in one session. These third-compartment fits were then used to test the hypothesis that the thirds of each compartment closest to the corridor are more globally coherent/less locally coherent than the thirds furthest away as they are closer together, such that there is reduced distance over which the path integrator may accumulate error. In the first five sessions, when the grids displayed a local representation, a paired, one-tailed t-test was used to ask whether the thirds furthest from the corridor were significantly more local than the thirds closest to the corridor. In contrast, in the last five sessions when the grids displayed a global representation, a paired, one-tailed t-test asked whether the grid patterns were more globally coherent in the thirds closest to rather than furthest from the corridor.

iv. Analysis of head direction cells

To analyse whether the activity of head direction cells disambiguated the two compartments, we calculated the angle of directional tuning and peak firing rate of head direction cells separately for the two compartments. Directional firing rate maps were calculated as described above, using positional and spiking data from either the left or right compartments. We then produced an angular cross-correlogram between the two directional firing rate maps for each cell by rotating one ratemap relative to the other and calculating the correlation between the two at each offset. To calculate the degree of angular offset between the directional tuning in each compartment we calculated the distance to the closest positive peak of the cross-correlogram from the origin, such that an offset of 0 indicates the highest correlation in the cross-correlogram occurred with no rotation of either directional ratemap. Angular offsets were then plotted as a function of experience of the multicompartment environment. The correlation between angular offset and recording

session was used to assess whether head direction cells disambiguated the two compartments with increasing experience. We next calculated the firing rate at the peak of directional tuning for the left and right compartments separately. A paired Wilcoxon signed-rank test was used to analyse whether head direction cell firing rates disambiguated the two compartments.

v. Phase offset analysis

The conclusions drawn from the fitting of ideal local and global grids were verified through a separate analysis of observed grid phase. In particular, we identified the difference in grid phase between the observed firing patterns in the right-hand compartment and that predicted from observed firing patterns in the left-hand compartment extended under a local or global representation. First, an ideal grid was fitted to the firing rate map recorded in the left-hand compartment of each grid cell according to the fitting principles described above. The ideal fitted grid was used to analyse the scale, orientation and phase of the recorded grid in the left compartment. Predictions were then made using the identified grid parameters about the location of grid fields in the right-hand compartment expected under a local or global firing pattern. That is, the local model predicts that firing fields replicate in the two compartments, so the phase in the right-hand compartment should equal the left. In the global model a single grid spans both compartments, thus the phase in the left compartment was 'projected' out to identify the phase in the right-hand compartment such that placed adjacent to one another a single grid spans both compartments. An ideal grid was then fitted to the firing pattern recorded in the right-hand compartment. Thus the identified phase of the recorded data could be compared to the phase predicted under the local and global models. The reported 'phase error' between the data and local or global models was then calculated as the magnitude of the vector connecting the observed and predicted phases in grid phase-space, and collapsed across all cells recorded in the same animal in the same session. Grid cells were again discarded where the previously calculated independent fit was <0.45. Further, the 10 data points where the predictions made by the local and global model

were most similar were also discarded to remove cells in which the models could not be distinguished. This equated to (18.52% of the 54 collapsed data points with an independent fit >0.45).

vi. Corridor grid cell classifier

The long and thin shape of the corridor meant analysis of the hexagonality of grid cell firing using a spatial autocorrelogram was unfeasible due to only sampling a small number of fields. To assess whether firing in the corridor was 'grid-like' we therefore used a grid cell classifier developed for analysing grid cells based on 1D linear-track firing [S5]. A smoothed firing rate map of the corridor of the multicompartment environment was first calculated. For each 2x2cm bin, a shuffled distribution of firing rates was calculated by shifting the positional information relative to spike times by at least 20 seconds and recalculating the firing rate in each bin for each of the 1000 shuffles. For each bin the proportion of shuffles which have a firing rate greater than that of the original firing rate map was then calculated. These proportions therefore allowed identification of bins in the original ratemap in which firing rates were higher or lower than would be expected by chance. Firing fields were defined as areas of at least 20 contiguous bins in which firing rates were greater than the 85th percentile of the bootstrapped shuffle distribution for those bins. Out of field regions were defined as areas with at least 20 contiguous bins where firing rates were less than the 5th percentile of the shuffled distribution. Cells were then classified as grid cells if: i. the corridor contained at least 3 firing fields as well as at least one out of field region. ii. 30% or more of the bins were assigned to either in-field or out-of-field areas. iii. The mean in-field to out-of-field firing rate ratio was greater than 2. The proportion of grid cells recorded in each day which satisfied these criteria was then plotted as a function of experience of the environment.

References:

S1. Barry, C., Hayman, R., Burgess, N. & Jeffery, K. J. (2007). Experience-dependent rescaling of entorhinal grids. *Nat. Neurosci.* **10,** 682–684.

S2. Kadir SN, Goodman DFM, Harris KD. (2014). High-dimensional cluster analysis with the masked EM algorithm. *Neural Computation* **26**:11.

S3. Sargolini, F., Fyhn, M., Hafting, T., McNaughton, B.L., Witter, M.P., Moser, M.-B., Moser, E.I. (2006). Conjunctive representation of position, direction, and velocity in entorhinal cortex. *Science* **312**, 758–762.

S4. Hafting, T., Fyhn, M., Bonnevie, T., Moser, M.-B., Moser, E.I. (2008). Hippocampus-independent phase precession in entorhinal grid cells. *Nature* **453** 1248-1242.

S5. Domnisoru, C., Kinkhabwala, A.A., Tank, D.W. (2013). Membrane potential dynamics of grid cells. *Nature* **495**, 199-204.

S6. Stensola, H., Stensola, T., Solstad, T., Froland, K., Moser, M.-B. & Moser, E.I. (2012). The entorhinal grid map is discretized. *Nature* **492** 72-78.
