# Supplementary figures and images for: Grid Cells Form a Global Representation of Connected Environments
Source: Curr Biol. 2015 May 4;25(9):1176–82. doi: 10.1016/j.cub.2015.02.037 (PMC4425461; doi:10.1016/j.cub.2015.02.037)

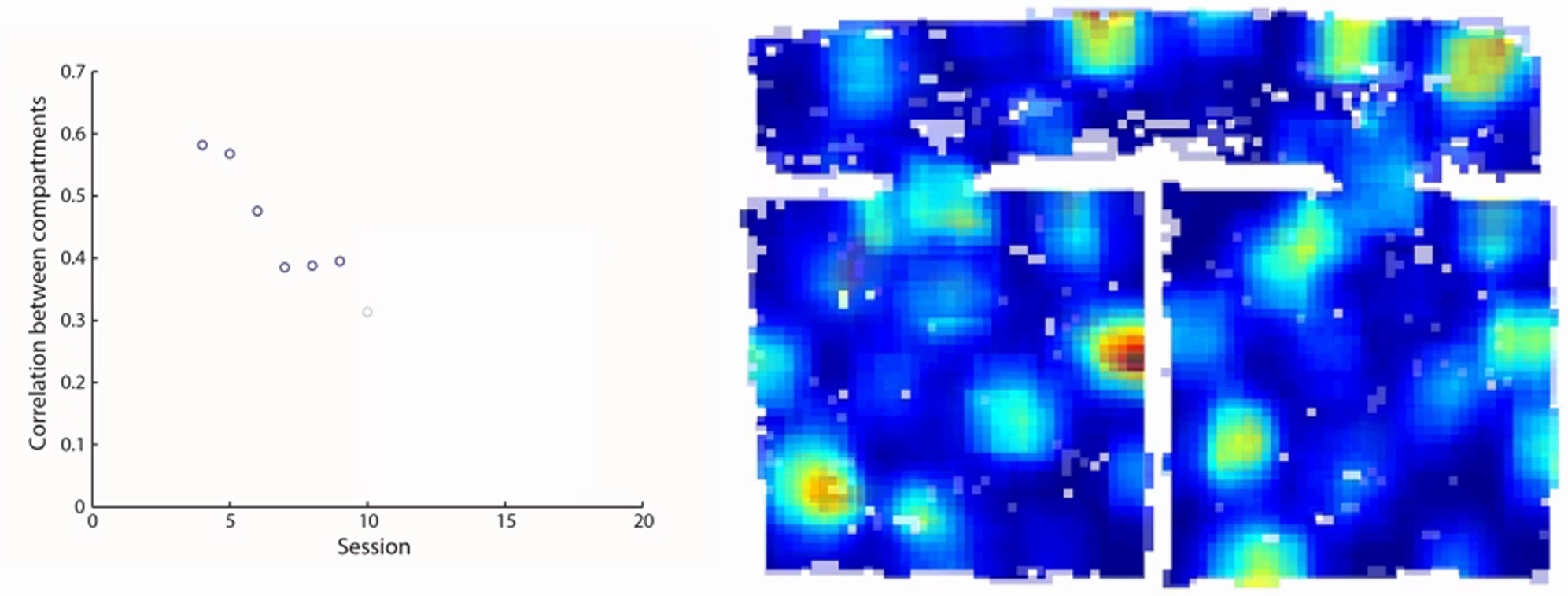

Supplement: Movie S1. The Firing Fields of a Single Grid Cell Recorded across 15 Sessions Appear to Shift Gradually and Continuously Rather Than Abruptly, Related to Discussion — Here, in the right-hand panel, the firing rate maps of a single grid cell recorded across 15 sessions are shown sequentially. Each rate map is the recorded firing of the cell in the second trial of each session. In the left-hand panel, the spatial correlation of firing rates in equivalent bins of the two compartments is plotted as a function of experience of the environment. Each data point is the spatial correlation for that trial alone. [file mmc2.jpg]
