# Supplementary material for: Grid Cells Form a Global Representation of Connected Environments
Source: Curr Biol. 2015 May 4;25(9):1176–82. doi: 10.1016/j.cub.2015.02.037 (PMC4425461; doi:10.1016/j.cub.2015.02.037)
Supplement: Document S2. Article plus Supplemental Information [file mmc3.pdf]

Report

Current Biology

Grid Cells Form a Global Representation of Connected Environments

Graphical Abstract

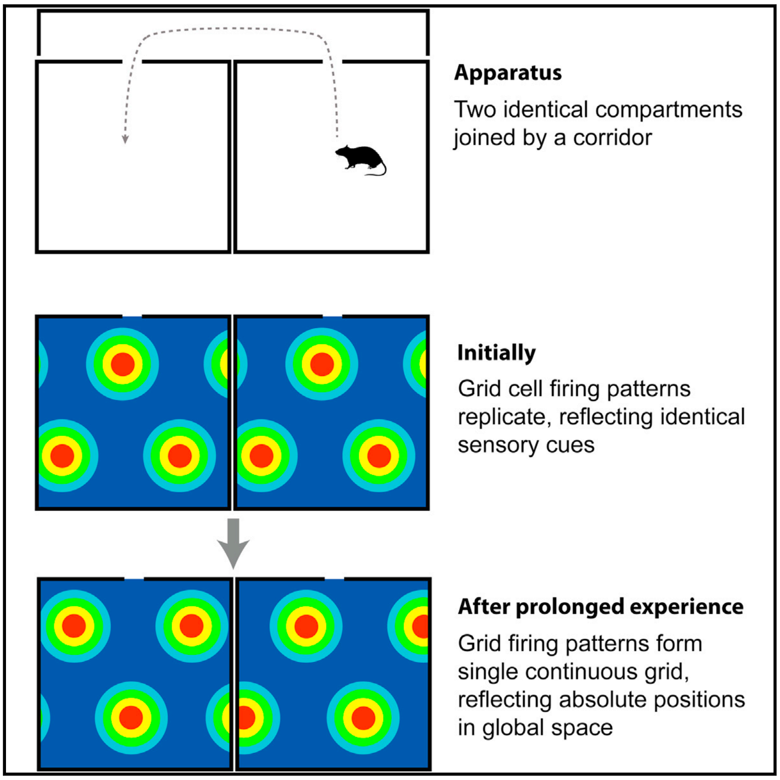

Highlights

- Grid cells were recorded in connected, perceptually identical compartments
- Initial grid firing patterns replicated between the two compartments
- With experience, a single, continuous grid pattern spanning both compartments formed
- This globally coherent pattern allows grid cells to act as a spatial metric

Authors

Francis Carpenter, Daniel Manson, ..., Neil Burgess, Caswell Barry

Correspondence

francis.carpenter.12@ucl.ac.uk (F.C.),
caswell.barry@ucl.ac.uk (C.B.)

In Brief

Grid cells are thought to provide an efficient metric for spatial navigation. However, this requires regular, continuous grid firing patterns across the environment. Carpenter et al. show that grid patterns are initially determined by sensory cues but self-correct with experience to form the globally coherent pattern required of a spatial metric.

Carpenter et al., 2015, Current Biology *25*, 1176–1182
May 4, 2015 ©2015 The Authors
http://dx.doi.org/10.1016/j.cub.2015.02.037

Current Biology
Report

Grid Cells Form a Global Representation of Connected Environments

Francis Carpenter,[1,2,*] Daniel Manson,[2,3] Kate Jeffery,[4] Neil Burgess,[1,5] and Caswell Barry[2,*]
[1]Institute of Neurology, UCL, Queen Square, London WC1N 3BG, UK
[2]Research Department of Cell and Developmental Biology, UCL, Gower Street, London WC1E 6BT, UK
[3]Centre for Mathematics and Physics in the Life Sciences and Experimental Biology, UCL, Gower Place, London WC1E 6BT, UK
[4]Institute of Behavioural Neuroscience, UCL, Bedford Way, London WC1H 0AP, UK
[5]Institute of Cognitive Neuroscience, UCL, Queen Square, London WC1N 3AR, UK
*Correspondence: francis.carpenter.12@ucl.ac.uk (F.C.), caswell.barry@ucl.ac.uk (C.B.)
http://dx.doi.org/10.1016/j.cub.2015.02.037
This is an open access article under the CC BY license (http://creativecommons.org/licenses/by/4.0/).

SUMMARY

The firing patterns of grid cells in medial entorhinal cortex (mEC) and associated brain areas form triangular arrays that tessellate the environment [1, 2] and maintain constant spatial offsets to each other between environments [3, 4]. These cells are thought to provide an efficient metric for navigation in large-scale space [5–8]. However, an accurate and universal metric requires grid cell firing patterns to uniformly cover the space to be navigated, in contrast to recent demonstrations that environmental features such as boundaries can distort [9–11] and fragment [12] grid patterns. To establish whether grid firing is determined by local environmental cues, or provides a coherent global representation, we recorded mEC grid cells in rats foraging in an environment containing two perceptually identical compartments connected via a corridor. During initial exposures to the multicompartment environment, grid firing patterns were dominated by local environmental cues, replicating between the two compartments. However, with prolonged experience, grid cell firing patterns formed a single, continuous representation that spanned both compartments. Thus, we provide the first evidence that in a complex environment, grid cell firing can form the coherent global pattern necessary for them to act as a metric capable of supporting large-scale spatial navigation.

RESULTS

We investigated whether grid cell firing patterns are determined by local sensory cues or whether they provide a coherent global representation of space by recording from 85 medial entorhinal cortex (mEC) grid cells in eight rats as they foraged within an environment containing two perceptually identical compartments connected via a corridor (Figure 1A). The environment was painted matte black and lit only by single lights on the south wall of each compartment. Black curtains encircled the environment to reduce the availability of distal cues. Each recording session consisted of two 40-min trials, with the floor of the environment rotated and the positions of the compartments swapped between trials to control unidentified sensory cues distinguishing the compartments. The environment therefore closely matched environments in which perceptual consistency between adjacent compartments has been shown to cause place cell firing fields to replicate [13–15]. We hypothesized that if grid cell representations are dominated by sensory cues, their firing should replicate between the two compartments. Conversely, if grid cell activity is determined by the global spatial features of the environment, their firing patterns should distinguish the two compartments due to their different absolute positions in space.

During early sessions, periodic firing patterns typical of grid cells were present in the environment and were replicated between the two compartments (Figure 1B). However, with increasing experience, the similarity of the representations between the two compartments decreased (Figure 1C), apparent in a negative correlation between the session number and the spatial correlation of firing rates between the two compartments ($r = -0.6674$, $r^2 = 0.4455$, $p = 1.669 \times 10^{-12}$; Figure 1D). The decrease in representation similarity across sessions was accompanied by an increase in hexagonal regularity of grid patterns within the compartments (Figure 1F). In the first five sessions, but not the last five sessions, gridness in the screening environment was greater than in the multicompartment environment (one-sample t tests, $t_{41} = 11.46$, $p = 2.328 \times 10^{-14}$ and $t_{25} = 0.8519$, $p = 0.4024$, respectively; Figure 1G), with the difference in gridness greater in the first than the last five sessions (unpaired t test, $t_{66} = 5.279$, $p = 1.443 \times 10^{-6}$).

To eliminate the possibility that unidentified local cues allowed disambiguation of the two compartments, we verified that firing was stable in global space and did not track the physical compartments when their positions were switched between trials. Specifically, in the last five sessions, the inter-trial spatial correlation between compartments in the same location was greater than the inter-trial spatial correlation between the same physical compartments in their new positions (paired t test, $t_{20} = 6.560$, $p = 2.160 \times 10^{-6}$; Figure 1E).

In contrast to grid cells, head direction cells continued to show the same directional tuning in the two compartments, regardless of experience (Figures S1A–S1C). The firing of a single border

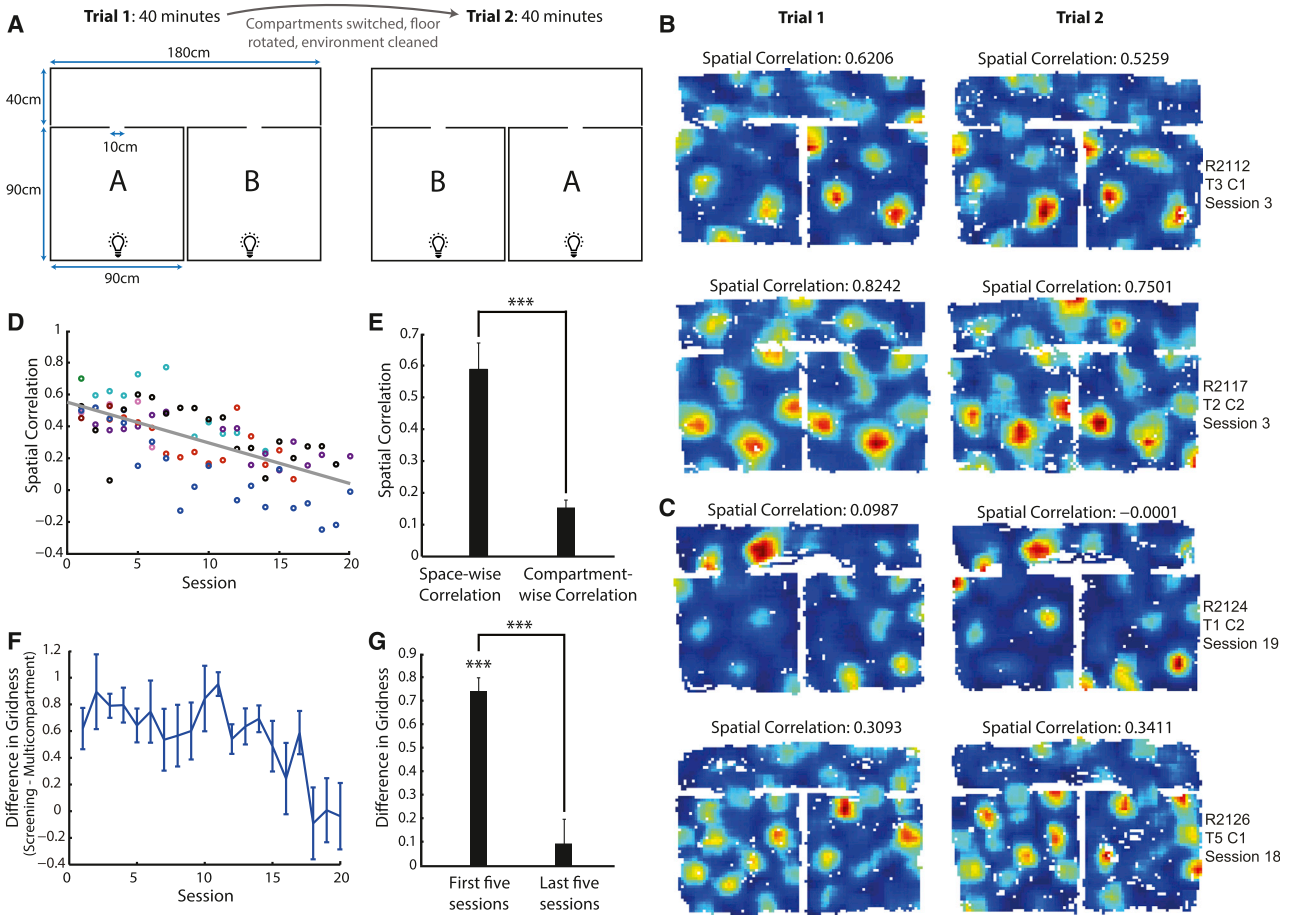

Figure 1. With Increasing Experience, Grid Cell Firing Patterns Show Reduced Representation Similarity between Compartments and Increased Regularity

(A) Schematic representation of the multicompartment environment and protocol for each recording session.

(B and C) Example firing rate maps. The left and right rate maps in each row are the same cell recorded in trial 1 and trial 2, respectively. Hotter colors indicate higher firing rates; unvisited bins are white. The correlation values above each plot are the spatial correlations of firing rates between the two compartments.

(B) Example grid cells recorded during early exposures to the multicompartment environment, where firing fields replicated between compartments.

(C) Example grid cells from late recording sessions, where firing patterns distinguished the compartments.

(D) Spatial correlations of grid cell firing rates between the compartments as a function of the animals' experience of the environment. Each data point represents the average correlation across all cells from one animal in one session, with different animals plotted in different colors.

(E) Spatial correlations between grid cell firing in equivalent absolute locations in successive trials ("space-wise correlation": e.g., compartment A trial 1 versus compartment B trial 2) or between equivalent locations within the same physical compartment in successive trials ("compartment-wise correlation": e.g., compartment A trial 1 versus compartment A trial 2), showing mean + SEM for cells in the last five sessions.

(F) The difference in gridness of firing patterns between the familiar square screening environment and the average of the gridness in each compartment (screening gridness − multicompartment gridness) as a function of experience. Plotted values are mean ± SEM across all cells recorded in each session.

(G) Difference in gridness (screening gridness − multicompartment gridness) in the first and last five sessions, showing mean and SEM; ***$p < 0.001$.

cell recorded in a late session also replicated between the two compartments (Figures S1D–S1F).

To determine whether grid firing in the two compartments predominantly reflected a local or global reference frame, we fitted ideal grid patterns to the recorded firing rate maps according to three models (see Supplemental Experimental Procedures for details). The grids were first fit by the "independent" model, in which grid phase was allowed to vary freely between the two compartments, while orientation and scale were required to be consistent (see Figures S2A–S2D). The independent fit represented the best possible fit of an ideal grid pattern to the data and was used to exclude grids too irregular to be well fit by any model, a necessary step given the reduced gridness seen in the multicompartment environment, particularly during early sessions (Figure 1F). The independent fit also determined the scale and orientation used in the "local" and "global" models. In the local model, the fitted grid had the same phase in each compartment, such that the firing fields replicated. The global model required the phase to be continuous across the compartments and so formed a single grid spanning the two. Local and global fits were normalized by the independent fit to allow comparison across cells. Firing patterns in the corridor were spatially stable and "grid-like"—consisting of peaks and troughs in firing. However, they were significantly less regular than firing

patterns in the compartments and were discarded from further analyses (Figures S3A–S3C). The irregularity was likely caused by the stereotyped behavior displayed by rats in the corridor (Figures S3D–S3F) and is consistent with past recordings of grid cells in linear environments [16, 17].

During early exposures, grid cell firing in the two compartments was best described by the local model (Figure 2A). However, with increasing experience, the local model's fit to the data decreased ($r = -0.5913$, $r^2 = 0.3496$, $p = 2.503 \times 10^{-6}$; Figure 2C). In contrast to the local model, the fit to the global model increased with experience, showing a positive correlation ($r = 0.4187$, $r^2 = 0.1753$, $p = 0.0016$; Figures 2B and 2D). A two-way ANOVA revealed an interaction between experience of the environment and the goodness of fit of the two models (session × model, $F_{(19,68)} = 1.89$, $p = 0.0293$; Figure 2E). We assessed whether in the first and last five sessions the local or global models fitted the data significantly better than would be expected under the null hypothesis of no particular phase relationship between the grids in each compartment. Specifically, each recorded rate map was fitted by 1,000 ideal grids with random phase offsets between the two compartments. For each cell/session conjunction with an independent fit >0.45, we then calculated the proportion of the 1,000 grids with random phase offsets which achieved a better fit than the local and global models. If no particular phase relationship between the grids in each compartment existed, the local and global models would on average fall in the middle of the distribution of the randomly offset grids. In contrast, in the first five sessions, the proportion of the 1,000 grids with a better fit than the local model was significantly lower than 0.5 (Wilcoxon signed-rank test [WSRT], $z = -3.724$, $p = 1.964 \times 10^{-4}$; Figure 2F). However, in the last five sessions, the local model no longer fit the data better than would be expected by chance (WSRT, $p = 0.6377$; Figure 2F). Conversely, in the last five sessions, but not the first five sessions, the global model's fit to the data was significantly better than expected from the null distribution (WSRTs, $p = 0.0019$ and $z = -0.1089$, $p = 0.9133$; Figure 2F). It is important to note that local and global representations are not mutually exclusive: grid patterns can be both identical in the two compartments and continuous across them both. As such, one would not necessarily expect consistently low local fits during late sessions or consistently low global fits during early sessions.

An independent analysis of the difference between observed grid phase in the right-hand compartment and that predicted from the left-hand compartment if firing formed a local or global representation confirmed a transition from a local to a global firing pattern (Figures S2G and S2H).

Grid cells are organized into functionally distinct modules [9, 18]. These modules are distributed non-uniformly in the brain, with modules in dorsal areas of mEC often having smaller-scale firing patterns than those found more ventrally [1, 18]. Here, as is typical of recordings in mEC, electrodes were implanted dorsally and advanced ventrally to locate grid cells. As such, the transition from a local to a global representation could be explained by biases in the sampling of modules across time. Specifically, if smaller-scale grid modules formed local representations and larger-scale modules formed global representations, the dorsal-to-ventral sampling bias could produce an artifactual local-to-global representation shift. To eliminate this possibility, we repeated the prior analysis separately for cells with scale either above or below the median grid scale. Both groups exhibited a significant shift from local to global representations (Figures S2E and S2F). However, as we did not record any grid cells with a scale less than the median in the last five sessions, we further analyzed the single grid scale (45–55 cm) for which grid cells with an independent fit >0.45 were recorded throughout the experiment. Again, the local model's fit decreased with experience ($r = -0.6185$, $r^2 = 0.3826$, $p = 0.0048$; Figure 3A), while the global model's fit increased ($r = 0.8001$, $r^2 = 0.6402$, $p = 3.902 \times 10^{-5}$; Figure 3B). Indeed, within this scale, grid firing patterns changed with experience from significantly more local to significantly more global than expected by chance between the first and last five sessions (Figure 3C). The same transition was also evident when analysis was restricted further to grid cells from a single module within an individual animal. In the animal with the most sequential recordings of grid cells from a single grid module and with an independent fit >0.45 (ten sessions), a two-way ANOVA revealed an interaction between experience of the environment and the goodness of fit of the two models (session × model, $F_{(8,25)} = 9.99$, $p = 0.0019$; Figures 3D and 3E), with the global model's fit increasing significantly with experience ($r = 0.7188$, $r^2 = 0.5167$, $p = 0.0056$; Figure 3E). That the transition from local to global representations is apparent within individual grid scales and modules demonstrates that it cannot be explained simply by biases in the sampling of grid scales and modules across time.

Grid cell firing likely derives from path integration: utilizing information about self-motion to update a representation of self-location [5, 19–22]. We therefore asked whether there was any difference in the grid representations between the thirds of each compartment closest to and furthest from the corridor. We hypothesized that the reduced distance between the sections of the compartments nearest the corridor may result in the accumulation of less path integration error and so produce more accurate global representations than in the sections furthest away. Confirming this hypothesis, in the first five sessions, grid patterns in the near third of each compartment were significantly less local than those in the furthest third (paired one-tailed t test, $t_{(17)} = -1.931$, $p = 0.0352$; Figure 3F), while in the last five sessions, grid patterns in the thirds closest to the corridor were significantly more global than those in the furthest thirds (paired one-tailed t test, $t_{(10)} = 1.959$, $p = 0.0392$; Figure 3F).

DISCUSSION

Grid cells are of great interest to computational neuroscientists as their periodicity allows them to form highly efficient spatial representations and to act as a metric for spatial calculations [5–7]. However, such calculations would be prone to significant errors where grid firing diverges from a regular and continuous pattern due to distortions and discontinuities. During initial exposures to the multicompartment environment, grid patterns were dominated by local sensory cues, replicating between the two compartments. However, with increasing experience, discontinuities in grid cell firing patterns between the compartments were incrementally reduced to form a single, continuous representation that spanned both compartments. This transition suggests grid cells adjust their firing to produce the globally coherent

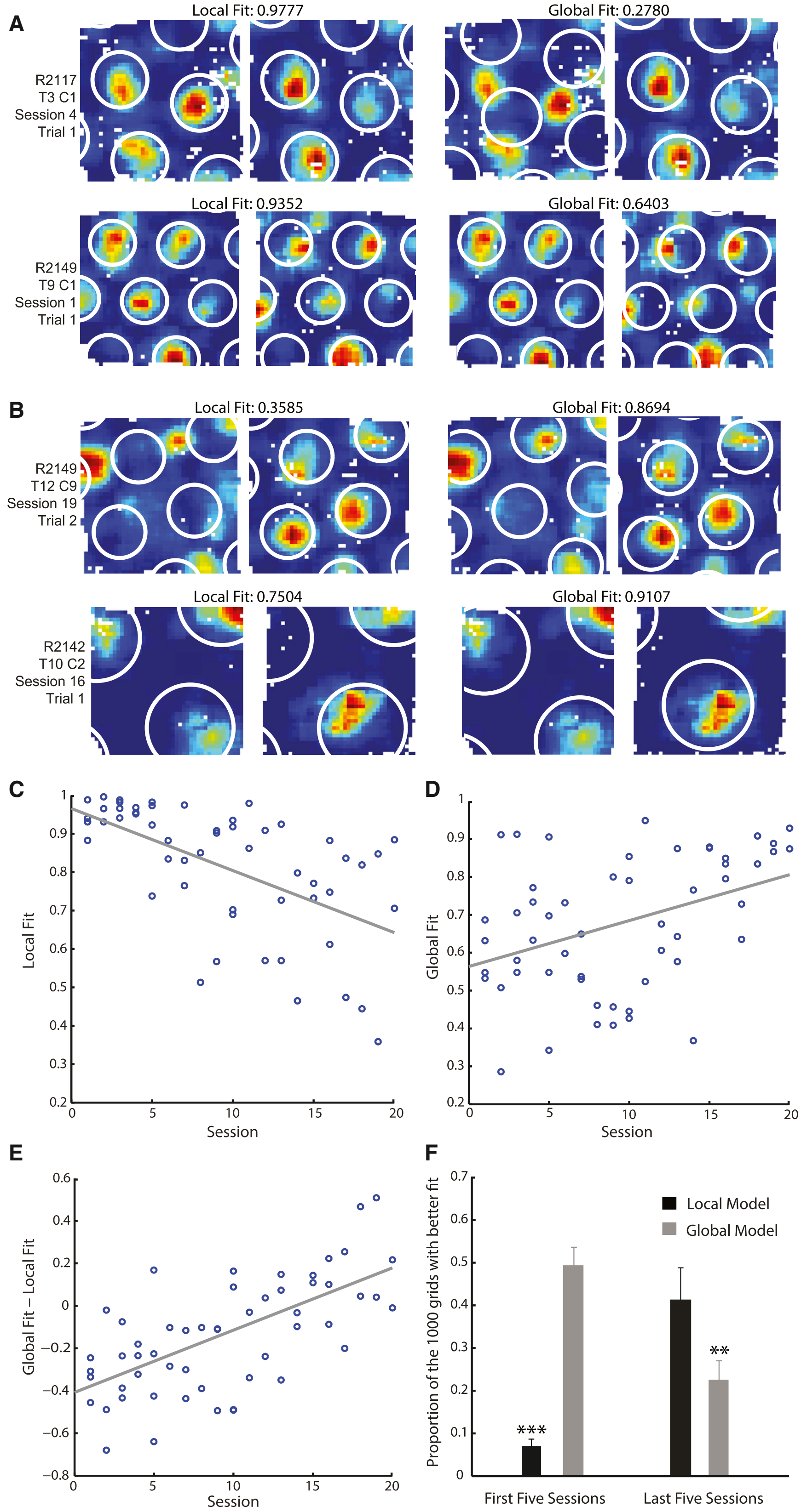

Figure 2. Grid Cell Firing Patterns Transition from a Local to a Global Representation with Increasing Experience

(A and B) Fits of local and global models to grid cell firing patterns in the two compartments. The local model was an ideal grid constrained to replicate between the two compartments, whereas the global model was a single continuous grid spanning both compartments. Each row is one cell in one trial: the underlying rate maps in the left and right columns are the same. The white rings overlaid indicate the best fitting local and global models in the left and right columns, respectively. Fit values show the spatial correlations between the local or global models and the data, normalized by the independent model's fit.

(A) Examples of grid cells recorded during early sessions, where the local model best fit the data.

(B) Example grids recorded during late sessions, where the global model best fit the data.

(C and D) The fit between grid cell firing patterns and ideal local and global grids, respectively, as a function of experience of the environment.

(E) The difference in the fit (global fit − local fit) between the global and local models across sessions.

In (C), (D), and (E), each data point represents the average fit for all cells with an independent fit >0.45, recorded from one animal in one session.

(F) The proportion of 1,000 ideal grids, with random phase offsets between compartments, with a better fit to the data than the local or global models. Values are mean + SEM across all cells with an independent fit >0.45 in the first or last five sessions. Wilcoxon signed-rank tests (WSRTs) compare observed values to an expected median of 0.5. **$p < 0.01$; ***$p < 0.001$.

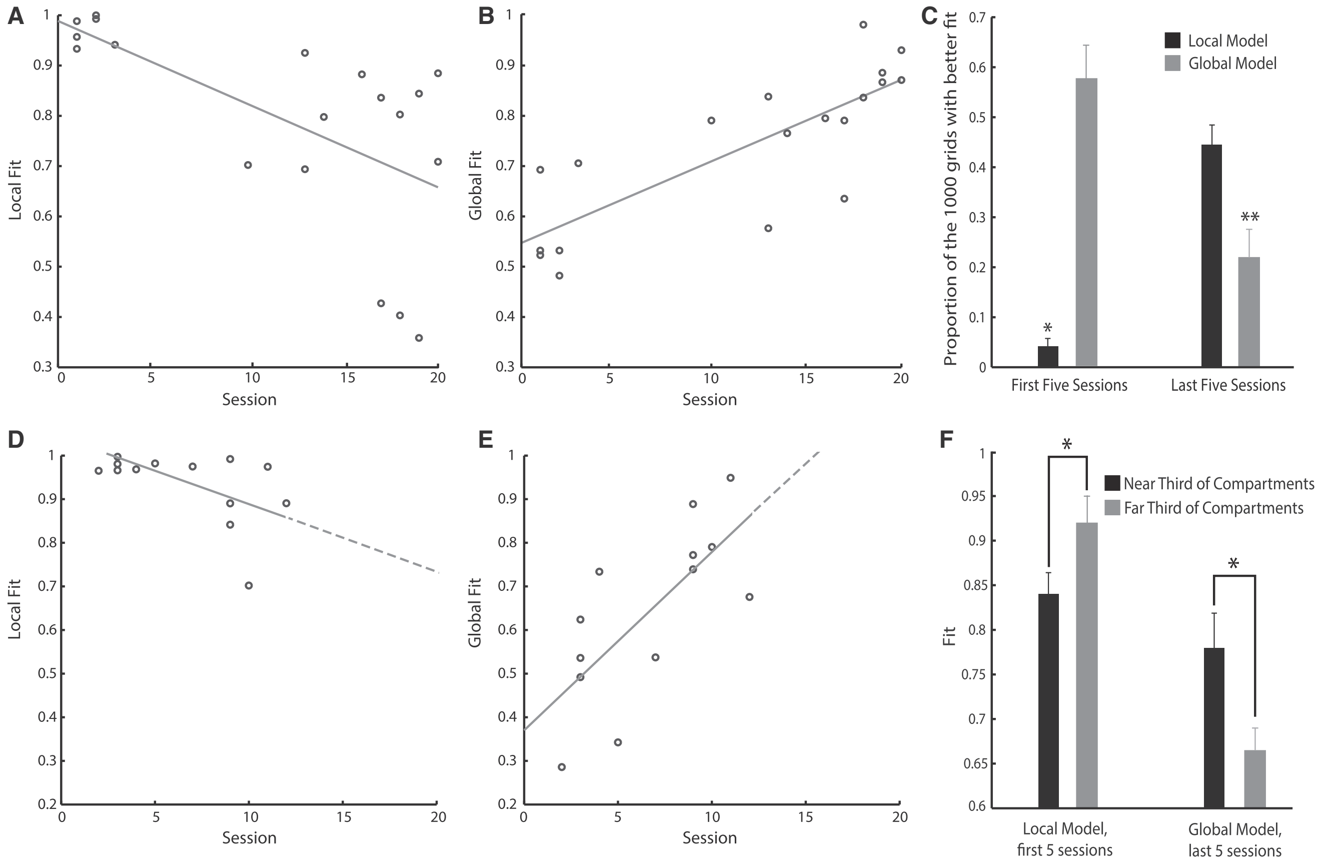

Figure 3. The Transition from Local to Global Representations Cannot Be Explained by Biases in the Sampling of Grid Cells

(A and B) The fit between recorded firing patterns of grid cells of a single scale and ideal local and global grids, respectively, as a function of experience of the environment. Only cells with a scale of 45 to 55 cm in the screening environment are included. Each data point represents the average local and global fits across all 45–55 cm cells with an independent fit >0.45, recorded from one animal in one session.

(C) The proportion of 1,000 ideal grids, with random phase offsets between the compartments, with a better fit to the cells in (A) and (B) than the local or global models. Values are mean + SEM across 45–55 cm cells with an independent fit >0.45 in the first or last five sessions. WSRTs compare observed values to an expected median of 0.5.

(D and E) The fit between recorded firing patterns of grid cells from a single module in a single animal and ideal local and global grids, respectively, as a function of experience. Dashed lines extend the least-squares lines to predict local and global fits in unrecorded sessions.

(F) The best fit achieved by the local model in the first five sessions and the global model in the last five sessions to the grid patterns in the thirds of the compartments nearest to or furthest from the corridor. Values are mean + SEM of the collapsed average within animals of cells with an independent fit >0.45. Paired, one-tailed t tests test whether difference in observed means differs from an expected mean of 0. *$p < 0.05$; **$p < 0.01$.

representation required for them to act as an effective spatial metric. Though the mechanism underpinning this transition remains undetermined, for grid cells to form a coherent global representation, it is necessary for them to identify the relative positions of all points in the environment. Models of grid cell formation largely describe their firing in terms of self-motion [5, 19–22], and the integration of self-motion as the animal explores the environment (path integration) is one way in which the relative position of points in space can be discerned. The more globally coherent firing patterns observed in the sections of the compartments closest together gives credence to this explanation: if the global coherence of grid patterns did not depend on path integration, one would not expect any difference across the compartments. The highly extended time frame over which cells transitioned from a local to a global representation made the continual recording from single cells across the whole period difficult. However, single grid modules, which are thought to form functional units [4, 9, 18], appeared to adjust their representations gradually and continuously (Figures 3D and 3E). Further, the firing fields of one grid cell recorded across 15 consecutive sessions appeared to shift continuously, rather than undergo a sudden transformation (Movie S1). These observations argue against an abrupt change in representation. However, to fully understand the temporal dynamics of the change from a local to a global representation, one would have to follow the same population of cells throughout the transition. The grid cells' gradual disambiguation of the compartments across sessions was reminiscent of the slow transition of place fields in morphed environments from boundary-referenced replication to direct remapping [23, 24], a process likely indicative of a slow plasticity-based mechanism [24, 25]. The common slow transition among grid and place cells likely indicates a unifying

underlying mechanism, though it remains to be seen whether changes in grid cell firing preempts or indeed drives changes in place cell firing.

EXPERIMENTAL PROCEDURES

For the full experimental protocol, please see Supplemental Experimental Procedures.

Animals and Surgery

All work was carried out under the Animals (Scientific Procedures) Act 1986 and according to Home Office and institutional guidelines. One or two microdrives were implanted above mEC in eight male Lister-Hooded rats.

Electrophysiological Recording and Experimental Protocol

Each session began with a 20-min baseline trial in which electrophysiological and positional data were acquired while animals foraged in a 1 × 1 m environment. Following identification of grid cells, rats were recorded while foraging in the multicompartment environment. Comprising two 90 × 90 × 50 cm compartments connected by a 180 × 40 × 50 cm corridor, the multicompartment environment was designed such that the two adjacent compartments would be as perceptually identical as possible.

Each recording session consisted of two 40-min trials, with the compartments' positions switched and the floor of the environment rotated between trials to control unidentified sensory cues distinguishing the compartments. Animals ran at most one session per day for a maximum of 20 sessions.

Analyses

Spike Sorting, Binning, and Grid Cell Inclusion Criteria

Spike sorting was performed offline using the automated clustering algorithm KlustaKwik [26].

Two-dimensional firing rate maps were calculated by assigning recorded positions and spikes to 2 × 2 cm bins covering the environment and dividing the number of spikes in each bin by the cumulative dwell time in each bin.

For inclusion in subsequent analysis, putative grid cells were first assessed using a gridness measure [27]. Cells were considered grid cells if their gridness exceeded the 99th percentile of a shuffled distribution of 1,000 gridness scores calculated from rate maps where spike times were randomly offset relative to position by at least 20 s. 85 cells passed these criteria, with a mean gridness of 0.89 in the baseline environment.

Correlations

To measure the similarity of grid firing between the two compartments, we calculated a Pearson product-moment correlation coefficient comparing firing rates in equivalent bins in the rate maps of the two compartments.

Fitting of Ideal Grids

Recorded rate maps were fitted by ideal grid patterns to determine whether grid representations were referenced to local or global features of the environment. In the local model, recorded rate maps were fitted by ideal grids whose firing patterns replicated between the two compartments. In contrast, ideal grids fitted in the global model consisted of a single grid pattern spanning both compartments. The less constrained independent model allowed identification of the best possible fit between each recorded rate map and any ideal grid. The independent model was therefore used to discard cells too irregular to be well fit by any model and to normalize fits in the local and global models.

SUPPLEMENTAL INFORMATION

Supplemental Information includes Supplemental Experimental Procedures, three figures, and one movie and can be found with this article online at http://dx.doi.org/10.1016/j.cub.2015.02.037.

AUTHOR CONTRIBUTIONS

F.C. collected the data. F.C., N.B., and C.B. analyzed the data and wrote the paper. D.M. contributed to data collection. F.C., K.J., N.B., and C.B. conceived and designed the experiment.

ACKNOWLEDGMENTS

This work was supported by the Wellcome Trust, UK Medical Research Council, and the Royal Society, as well as by E.U. SpaceCog and Human Brain Project grants to N.B.

Received: December 15, 2014
Revised: January 22, 2015
Accepted: February 12, 2015
Published: April 23, 2015

REFERENCES

1. Hafting, T., Fyhn, M., Molden, S., Moser, M.-B., and Moser, E.I. (2005). Microstructure of a spatial map in the entorhinal cortex. Nature *436*, 801–806.
2. Boccara, C.N., Sargolini, F., Thoresen, V.H., Solstad, T., Witter, M.P., Moser, E.I., and Moser, M.-B. (2010). Grid cells in pre- and parasubiculum. Nat. Neurosci. *13*, 987–994.
3. Fyhn, M., Hafting, T., Treves, A., Moser, M.-B., and Moser, E.I. (2007). Hippocampal remapping and grid realignment in entorhinal cortex. Nature *446*, 190–194.
4. Yoon, K., Buice, M.A., Barry, C., Hayman, R., Burgess, N., and Fiete, I.R. (2013). Specific evidence of low-dimensional continuous attractor dynamics in grid cells. Nat. Neurosci. *16*, 1077–1084.
5. McNaughton, B.L., Battaglia, F.P., Jensen, O., Moser, E.I., and Moser, M.-B. (2006). Path integration and the neural basis of the 'cognitive map'. Nat. Rev. Neurosci. *7*, 663–678.
6. Buzsáki, G., and Moser, E.I. (2013). Memory, navigation and theta rhythm in the hippocampal-entorhinal system. Nat. Neurosci. *16*, 130–138.
7. Fiete, I.R., Burak, Y., and Brookings, T. (2008). What grid cells convey about rat location. J. Neurosci. *28*, 6858–6871.
8. O'Keefe, J., and Nadel, L. (2008). The Hippocampus as a Cognitive Map. (Oxford University Press).
9. Barry, C., Hayman, R., Burgess, N., and Jeffery, K.J. (2007). Experience-dependent rescaling of entorhinal grids. Nat. Neurosci. *10*, 682–684.
10. Stensola, T., Stensola, H., Moser, M.B., and Moser, E.I. (2015). Shearing-induced asymmetry in entorhinal grid cells. Nature *518*, 207–212.
11. Krupic, J., Bauza, M., Burton, S., Barry, C., and O'Keefe, J. (2015). Grid cell symmetry is shaped by environmental geometry. Nature *518*, 232–235.
12. Derdikman, D., Whitlock, J.R., Tsao, A., Fyhn, M., Hafting, T., Moser, M.B., and Moser, E.I. (2009). Fragmentation of grid cell maps in a multicompartment environment. Nat. Neurosci. *12*, 1325–1332.
13. Skaggs, W.E., and McNaughton, B.L. (1998). Spatial firing properties of hippocampal CA1 populations in an environment containing two visually identical regions. J. Neurosci. *18*, 8455–8466.
14. Lever, C., Burgess, N., Cacucci, F., Hartley, T., and O'Keefe, J. (2002). What can the hippocampal representation of environmental geometry tell us about Hebbian learning? Biol. Cybern. *87*, 356–372.
15. Spiers, H.J., Hayman, R.M.A., Jovalekic, A., Marozzi, E., and Jeffery, K.J. (2015). Place field repetition and purely local remapping in a multicompartment environment. Cereb. Cortex *25*, 10–25.
16. Hafting, T., Fyhn, M., Bonnevie, T., Moser, M.-B., and Moser, E.I. (2008). Hippocampus-independent phase precession in entorhinal grid cells. Nature *453*, 1248–1252.
17. Domnisoru, C., Kinkhabwala, A.A., and Tank, D.W. (2013). Membrane potential dynamics of grid cells. Nature *495*, 199–204.
18. Stensola, H., Stensola, T., Solstad, T., Frøland, K., Moser, M.-B., and Moser, E.I. (2012). The entorhinal grid map is discretized. Nature *492*, 72–78.
19. O'Keefe, J., and Burgess, N. (2005). Dual phase and rate coding in hippocampal place cells: theoretical significance and relationship to entorhinal grid cells. Hippocampus *15*, 853–866.
20. Fuhs, M.C., and Touretzky, D.S. (2006). A spin glass model of path integration in rat medial entorhinal cortex. J. Neurosci. *26*, 4266–4276.

21. Burak, Y., and Fiete, I.R. (2009). Accurate path integration in continuous attractor network models of grid cells. PLoS Comput. Biol. *5*, e1000291.
22. Etienne, A.S., and Jeffery, K.J. (2004). Path integration in mammals. Hippocampus *14*, 180–192.
23. O'Keefe, J., and Burgess, N. (1996). Geometric determinants of the place fields of hippocampal neurons. Nature *381*, 425–428.
24. Lever, C., Wills, T., Cacucci, F., Burgess, N., and O'Keefe, J. (2002). Long-term plasticity in hippocampal place-cell representation of environmental geometry. Nature *416*, 90–94.
25. Barry, C., and Burgess, N. (2007). Learning in a geometric model of place cell firing. Hippocampus *17*, 786–800.
26. Kadir, S.N., Goodman, D.F.M., and Harris, K.D. (2014). High-dimensional cluster analysis with the masked EM algorithm. Neural Comput. *26*, 2379–2394.
27. Sargolini, F., Fyhn, M., Hafting, T., McNaughton, B.L., Witter, M.P., Moser, M.-B., and Moser, E.I. (2006). Conjunctive representation of position, direction, and velocity in entorhinal cortex. Science *312*, 758–762.

Current Biology

Supplemental Information

Grid Cells Form a Global Representation of Connected Environments

Francis Carpenter, Daniel Manson, Kate Jeffery, Neil Burgess, and Caswell Barry

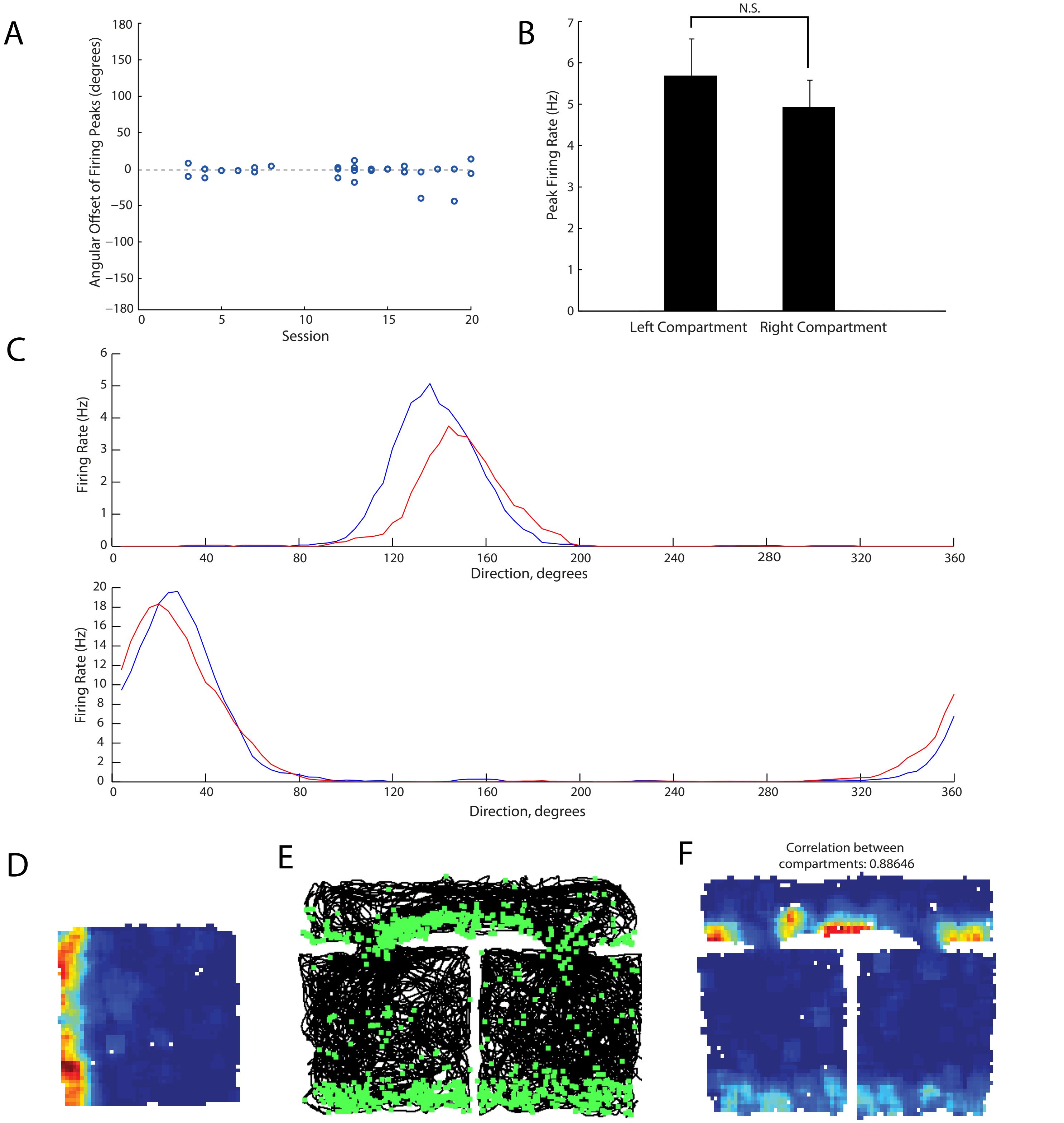

Supplementary Figure 1: Head direction and border cell firing replicates in the two compartments regardless of experience: Further data relevant to Figure 1.

A, B and **C**: Head direction cell firing replicated in the two compartments regardless of experience. **A**. The angular offset between the firing rate peaks of directional ratemaps from the left and right compartments. Angular offsets were calculated by producing a directional cross-correlogram between directional firing rate maps of the two compartments and calculating the distance from the origin to the cross-correlogram peak. For each recorded head direction cell offsets are plotted as a function of experience of the environment. A negative angular offset indicates the firing peak in the left compartment is anticlockwise of the firing peak in the right compartment. Peak offsets are clustered around 0º regardless of experience, and the correlation between peak offset and session was not significant (r = -0.1744, p = 0.3481). **B**. Peak directional firing rates were also consistent between the compartments. Plotted values are mean + sem (Wilcoxon signed rank test, z = 1.666, p = 0.0958). **C**. Example directional firing rate maps for two representative head direction cells recorded from two rats. The directional firing rate map of the left compartment is plotted in blue and the right compartment in red. **D, E** and **F**: A putative border cell recorded in session 11 showed replicated firing between the two compartments of the multicompartment environment. **D**. The firing rate map of a putative border cell recorded in the screening environment. Higher firing rates are indicated by hotter colours, unvisited bins are white. **E**. The unbinned path of the animal as it explores the multicompartment environment in black, overlaid in green with the location of spikes from the same cell as in **D**. **F**. Firing rate map of the same putative border cell. The cell was recorded in the 11th session, yet like head direction cells, even with extended experience, shows highly consistent firing in the two compartments.

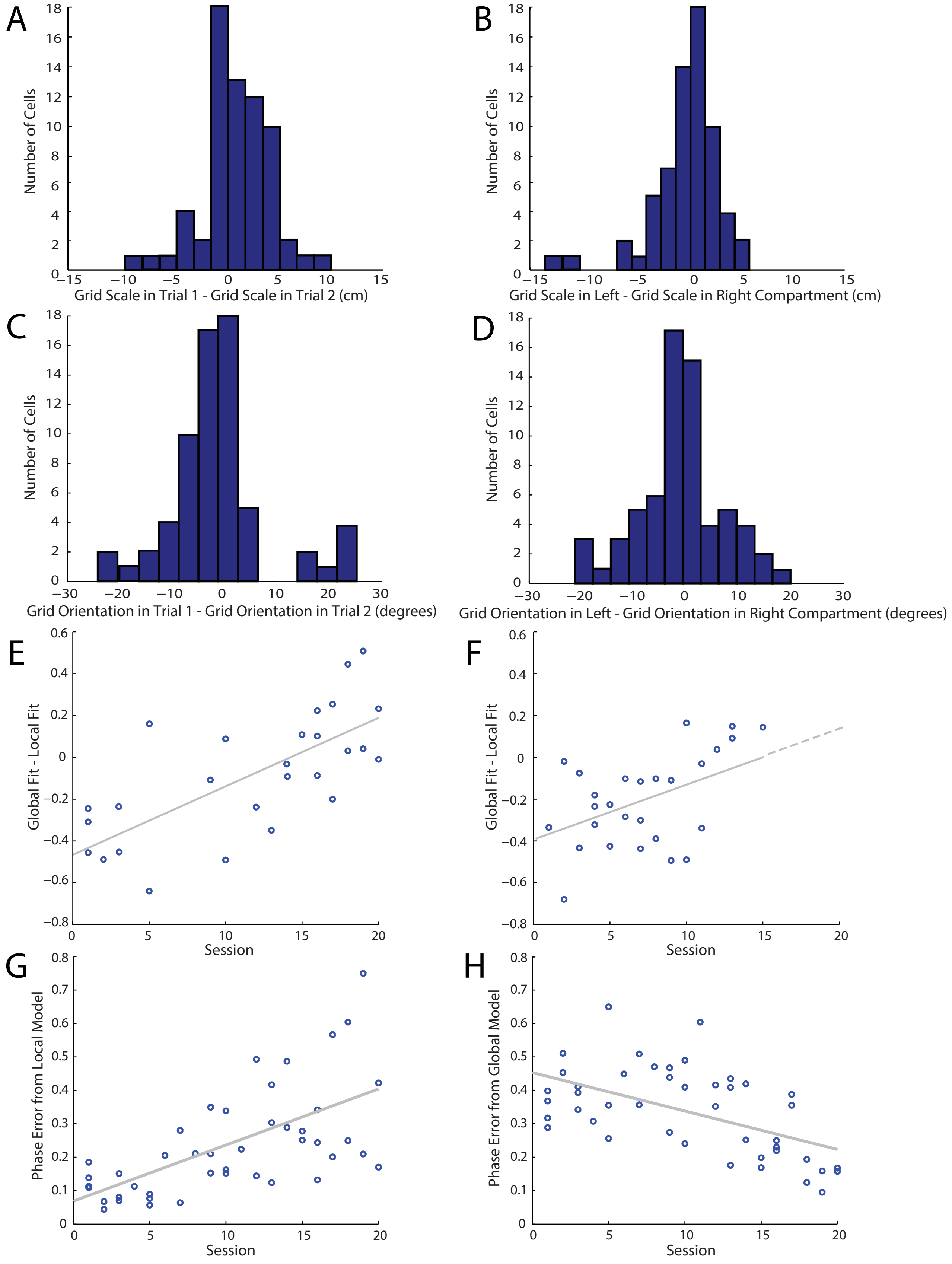

Supplementary Figure 2: Grid properties are consistent in the two compartments. Analysis of grid phase confirms local to global representation transition: Further data relevant to Figure 2.

A - D: Grid properties are consistent in the two compartments. For each grid cell recorded in both trials of a single session, the differences in grid scale (**A**, **B**) and orientation (**C**, **D**) between trial 1 and trial 2 and between the left and right compartments were calculated from each trial's spatial autocorrelogram. Only cells with a fit of the independent model of 0.45 were included to discard cells which were sufficiently irregular that estimates of grid properties were inaccurate. To analyse whether the difference in grid scale and orientation between compartments was greater than that between trials, Wilcoxon signed-rank tests compared the medians of the absolute (the magnitude of the) differences between trials and between compartments. The differences in grid scale between compartments and between trials were not significantly different (z = -0.7397, p = 0.4597). Neither did the differences in grid orientation differ significantly (z = 0.8982, p = 0.3691). **E** and **F**: both small and large scale grid cells transition from local towards global representations. In **E** and **F** the difference in fits between the global and local model (Global - Local) is plotted as a function of experience of the environment separately for grid cells with scale greater than the median grid scale (**E**) and for grid cells smaller than the median grid scale (**F**). A significant positive correlation is observed in both larger (r = 0.6862, r^2 = 0.4709, p = 0.0001) and smaller (r = 0.5057, r^2 = 0.2557, p = 0.0071) scale grid cells. Data points represent the average fit across all cells recorded in one animal in one session. **G** and **H**: Phase offset analysis confirms transition from a local to a global representation. In **G** and **H**, phase error equates to the magnitude of the vector in grid-phase space connecting the observed phase in the right-hand compartment to the phase predicted under local or global models based on grid parameters estimated from firing fields in the left-hand compartment (see Supplementary Methods). Each data point represents the average phase error for all cells recorded from one animal in one session. In **G**, the phase error from the local model increases significantly as a function of experience of the environment (r = 0.6274, r^2 = 0.3936, p = 5.179x10^{-7}), indicating the grids become less local with experience. In contrast, in **H** the phase error from the global model decreases across sessions (r = -0.5514, r^2 = 0.3040, p = 1.048x10^{-4}), confirming that grids become more global with experience.

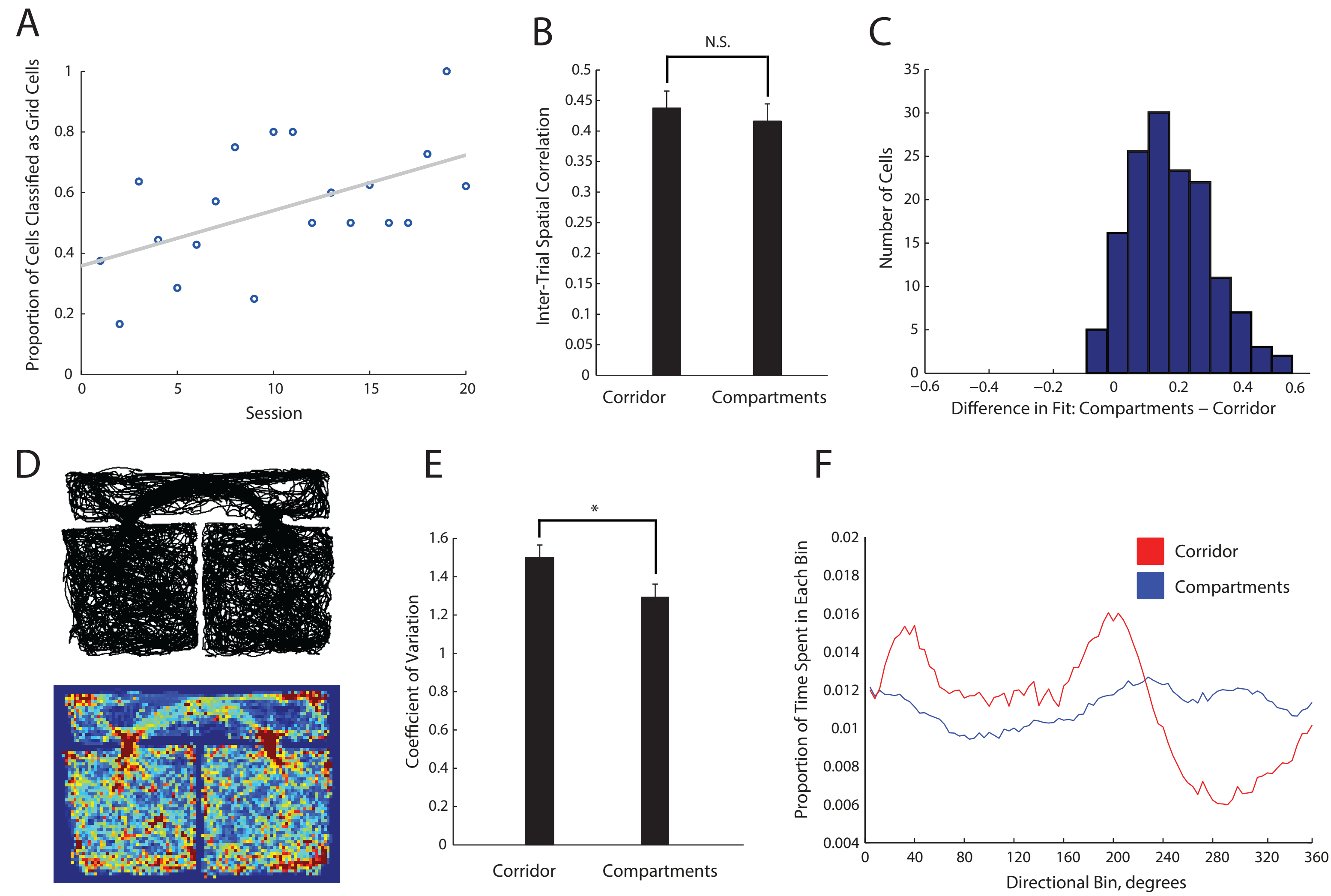

Supplementary Figure 3: Firing patterns in the corridor are 'grid-like' and stable, but irregular, co-occurring with highly stereotyped behaviour: Further data relevant to Figure 2.

A. The proportion of cells designated as grid cells by a Grid Cell Classifier analysing firing in the corridor (see Supplementary Methods) indicates the presence of grid-like firing in the corridor, which becomes more pronounced with experience of the environment (*r* = 5238, r^2 = 0.2744, *p* = 0.0177). **B**. The spatial-correlation of firing rates in trial 1 with firing rates in equivalent positions in space in trial 2, calculated separately for the compartments and corridor. Data are presented as mean + sem across all cells recorded in both trials of a single session. Firing was equally stable in the compartments and corridor (*paired t-test*, t_{114} = 1.278, *p* = 0.2037). **C**. The best fit achieved between ideal grids and recorded firing patterns in the corridor or compartments. Ideal grids fitted were required to have the same orientation and scale as was previously found to achieve the best independent fit. The difference in best fit between the corridor and compartments for all grid cells is plotted in a histogram. The best observed fit to firing in the corridor was significantly lower than that to the compartments (*unpaired t-test*, t_{143} = 14.52, *p* = 6.055x10^{-30}). **D**. Top: the path taken by an animal in a typical, 40 minute recording trial. Bottom: Heat map of unsmoothed dwell times for the same trial, hotter colours indicate longer dwell. **E**. The coefficient of variation ($C_v = \sigma / \mu$) of dwell time between bins, averaged across all recording trials. The coefficient of variation was significantly greater in the corridor than the compartments (*paired t-test*, t_{85} = 3.7328, *p* = 3.414x10^{-4}), indicating greater variability in dwell times between bins in the corridor than compartments. **F**. The mean proportion of time spent in each head direction bin across all animals in the corridor (red) and compartments (blue) differed significantly between the corridor and the compartments (*Kolmogorov-Smirnov test*, *p* = 4.352x10^{-4}). In the corridor large biases in head direction are evident, likely due to the ballistic running between the compartments evident in **D**. In the compartments behaviour is less stereotyped, and the animals sampled all spatial and directional bins more evenly.

Supplementary Methods:

Animals

All work was carried out within the terms of the Animals (Scientific Procedures) Act 1986, and according to Home Office and institutional guidelines. 8 experimentally naïve, male Lister Hooded rats (275-400g at surgery) were housed communally under a 12:12 inversed light-dark cycle for at least one week prior to surgery. Following surgery, animals were held individually in Perspex cages and restricted to 90% of their free-feeding body weight.

Microdrives and surgery

During surgery, rats received either 1 or 2 custom-made microdrives. Each microdrive consisted of 32 HML-coated 17-µm Platinum-Iridium (90-10%) electrodes twisted into 8 separate tetrodes. The electrodes were mounted on a single drive-mechanism (tetrodes not independently adjustable) which allowed for their advancement through the turning of a trapped screw. Before surgery electrode tips were electroplated in a Platinum solution to <150kΩ impedance.

Microdrives were implanted following the same surgical procedures as described previously [S1]. In four rats, a trephine was used to drill a single craniotomy above dorso-lateral medial entorhinal cortex (mEC). In two rats, craniotomies were drilled bilaterally above dorso-lateral mEC in both hemispheres. In the final two rats, craniotomies were drilled above mEC in one hemisphere and the Hippocampus (HPC) in the other. For mEC implants, electrodes were angled anteriorly 8-10° and implanted 4.5mm lateral to Bregma, 0.3mm anterior to the transverse sinus and at a depth of 1.6mm from the surface of the brain. Following surgery animals were allowed at least a one week recovery period in which Baytril (enrofloxacin – 4ml/100ml water) was given in the water to prevent infection and Metacam (meloxicam – 5mg/kg) suspended in jelly acted as an analgesic.

Electrophysiological recording and behavioural training

Electrophysiological and positional data was acquired using the DACQ (Axona Ltd., St Albans, UK) recording system, details of which are described in Barry et al. [S1].

Neural activity was recorded while animals were trained to forage for sweetened rice in a square 1mx1m environment comprising four 60cm high grey walls held together by corner clamps. The environment was placed on an earthed black metal-composite sheet in the centre of a room containing a number of distal landmarks to aid orientation. Both the walls and floor were cleaned between recording trials. Electrodes were advanced in 30-60µm steps between recordings until grid cells were identified.

Experimental environment and protocol

Each recording session began with a 20 minute baseline trial in the square screening environment. Following identification of grid cells, rats were placed in an enclosed white-plastic travel box while the experimental environment was prepared. Comprising two 90x90x50cm compartments connected by a 180x40x50cm corridor, the multicompartment environment was designed such that the two adjacent compartments would be as perceptually identical as possible. Painted matte black, the environment was placed on a transparent-Perspex floor on top of a uniform black plastic groundsheet, and surrounded by four black curtains organised in a square. Each compartment contained a light at the centre of the south wall, 40cm from the floor. Each light was a battery-powered bike light mounted to the outside of the compartment and enclosed in aluminium-foil. The light entered each compartment solely through a 3cm diameter window covered with translucent frosted-Perspex. No other light source was present. Into the north face of each compartment was cut a trapezoidal doorway, measuring 10cm across at its base and 50cm across at its top. Each compartment was reinforced along its base with a 1cm strip of stainless steel, allowing them to be easily moved without changing shape.

At the beginning of each experimental session the floor of the environment was cleaned. The first experimental trial was then started, with the rat being placed in the corridor between the two compartments, facing the north wall. Throughout the trial the experimenter moved pseudo randomly around the circumference of the environment, distributing rice such that the rat explored the whole arena. The tether of the recording system was counterbalanced and suspended above the animal using 'runners' such that the centre of mass of the tether moved freely as the animal did, ensuring no directional cue was provided. After 40 minutes, or when the rat had satisfactorily covered the entire environment, the rat was returned to its travel box. The compartments were then removed and the Perspex floor was rotated 180° before being cleaned. Once dry, the compartments were replaced in the opposite positions to the first trial, such that the compartment in the west of the environment during the first trial was now in the east and vice versa. The animal was then again placed in the corridor facing the north wall and the second experimental trial began. Rats ran at most one session per day for a maximum of 20 sessions. Recordings in the multicompartment environment continued on subsequent days as long as at least one grid cell was still identifiable during screening. If no grid cell was present, the electrodes were again moved until further grid cells were found.

Spike sorting and binning

Spike sorting was performed offline through fitting a mixture of Gaussians according to an expectation-maximisation algorithm using KlustaKwik [S2]. Putative clusters were then further analysed using the data analysis suite Tint (Axona Ltd., St Albans, UK). In particular, Tint was used to coordinate clusters across trials and to correct for over-clustering according to the amplitude, waveform and temporal autocorrelation of spikes.

For two-dimensional firing rate maps the animals' recorded positions and spikes were assigned to 2x2cm bins covering the environment. Unsmoothed firing rate maps were calculated by dividing the number of spikes assigned to each bin by the cumulative dwell time in each bin. Smoothed ratemaps

were constructed using a 5x5bin boxcar filter, with the firing rate in bin i equal to the number of spikes in the kernel centred on i divided by the occupancy of the kernel.

For directional ratemaps, recorded heading directions and spikes were assigned to 4° bins between 0 and 360°, with the animal's head direction calculated from the angular offset of two groups of LEDs on the animal's head-stage. Unsmoothed ratemaps were constructed by dividing the number of spikes by the cumulative dwell in each directional bin. The Kullback-Leibler Divergence (KL Divergence) was used to measure the divergence between the resulting circular ratemap and a uniform circular distribution. Cells were classified as head direction cells where the KL Divergence in the screening environment exceeded 0.25.

Spatial Autocorrelograms and Grid Cell Inclusion Criteria

For inclusion in subsequent analysis, putative grid cells were assessed using a gridness measure, following Sargolini et al. 2006 [S3]. For both the screening environment, and for each compartment of the multicompartment environment, a spatial autocorrelogram was constructed from the smoothed ratemap, according to:

$$r(\tau_x,\tau_y) = \frac{n\Sigma\lambda(x,y)\lambda(x-\tau_x,y-\tau_y) - \Sigma\lambda(x,y)\Sigma\lambda(x-\tau_x,y-\tau_y)}{\sqrt{n\Sigma\lambda(x,y)^2 - \left(\Sigma\lambda(x,y)\right)^2}\,.\sqrt{n\Sigma\lambda\left(x-\tau_x,y-\tau_y\right)^2 - \left(\Sigma\lambda\left(x-\tau_x,y-\tau_y\right)\right)^2}}$$

Where $r(\tau_x,\tau_y)$ is the autocorrelation between bins with spatial offset τ_x and τ_y. $\lambda(x,y)$ is the firing rate in bin (x,y), while n is the total number of bins. The six local maxima closest to but excluding the origin of the autocorrelogram were used to identify the orientation, scale and gridness of the putative grid cell. Orientation was measured as the angle from a consistent arbitrary horizontal reference line to the first peak of the autocorrelogram in an anticlockwise direction. The median distance from the origin to the six peaks was used to estimate the scale of the putative cell. Finally, the gridness was calculated by rotating the autocorrelogram in 30°steps for 150° and taking

the Pearson product-moment correlation coefficient between each rotated autocorrelogram and the un-rotated autocorrelogram. The maximal correlation obtained at 30, 90 or 150° was subtracted from the minimum correlation found at 60 or 120° to produce the gridness score. When analysing the gridness of firing patterns in the multicompartment environment, gridness scores were calculated separately for each compartment and subsequently mean averaged.

For each putative grid cell the gridness score obtained from the screening ratemap or the multicompartment ratemap was compared to the distribution of gridness scores observed following 1000 shuffles of the positional and spiking data of the same cell during the same trial. That is, for each shuffle the relative timing of the position and spike data was circularly shifted randomly by at least 20 seconds, with the autocorrelogram and gridness then calculated using the ratemap resulting from each shift. To be considered a grid cell and included in subsequent analysis, a cell was required to have a gridness score in the 99th percentile of the distribution of shuffled gridness scores in either the screening or multicompartment environment.

To ensure that no single grid cell recorded across tetrodes was considered two separate cells, where any putative cells with similar firing fields were observed within or between tetrodes a spatial cross-correlation was first performed. If the two putative grid cells had consistent spatial firing the temporal cross-correlation between the cells was subsequently analysed. Where pairs of putative grid cells had both high spatial and temporal cross-correlations they were considered to be single cells.

85 grid cells passed these inclusion criteria, with a mean average gridness of 0.89 in the screening environment.

Analyses

i. General

The 85 grid cells were recorded across 99 sessions. There were 59 instances of recording a cell in a session in which that cell had been recorded in at least one previous session. In total, there were 144 grid cell/session conjunctions (85 unique cells + 59 instances of re-recording). In 115/144 of the cell/session conjunctions the grid cell was held across both multicompartment trials. Here, any calculated metric is mean-averaged across the two trials, yielding a single value for every cell in each session.

Unless specifically analysing the corridor, all other analyses discarded the corridor and analysed only ratemaps formed by the left and right compartments separated by the gap created by the central wall. While firing in the corridor was grid-like - formed of peaks and troughs in firing (Supplementary Fig 3A), and stable (Supplementary Fig 3B), firing in the corridor was significantly less regular and hexagonal than the compartments (Supplementary Fig 3C). This irregularity is consistent with past recordings of grid cells in linear environments [S4, S5], and was likely exacerbated by highly stereotyped behaviour in the corridor: In the corridor animals tended to run in a ballistic fashion between the compartments, resulting in a highly uneven sampling of space and direction (Supplementary Fig 3D-F). As any ideal grid could only be poorly fitted to the corridor, the comparison of alternative models through the fitting of ideal grids was uninformative.

ii. Correlations

To measure the similarity of grid cell representation between the left and right compartments a Pearson product-moment correlation coefficient was calculated comparing the firing rates in equivalent bins of the smoothed ratemaps of the two compartments. Bins were discarded if they were unvisited in either compartment or they had a firing rate of 0 in both compartments.

Further correlations were used to assess whether grid cell representations tracked the movement of the compartments between trials. For each cell recorded across both trials in a single day Pearson product-moment correlation coefficients were calculated between firing rates in equivalent bins in the same compartment in successive trials (compartment-wise correlation), or between equivalent bins in the same position in global space in successive trials (space-wise correlation).

iii. Fitting of ideal grids

Ideal grid patterns were fitted to ratemaps according to three models to analyse whether the recorded grid representations could be best described as being determined by local or global features of the environment. The recorded grid firing patterns were first fit by the 'independent' model. Here, 900 ideal grids were generated, each the product of three cosine gratings with wave vectors k_1, k_2 and k_3 and phase offsets c_1, c_2 and c_3. Wave vector $\vec{k} = \left(\frac{2\pi}{\lambda}\cos(\varphi), \frac{2\pi}{\lambda}\sin(\varphi)\right)$ where λ is the grating wavelength ($\lambda = G\sqrt{3}/2$, where G is the grid scale), and φ is the grating orientation. The three wave vectors form a regular triangular grid where orientations differ by $\frac{2\pi}{3}$ (i.e., 120°) and $c_1 + c_2 = c_3$. As such, firing rate as a function of location is given by:

$$f(\vec{x}) = A\left(1 + \cos\left(\overrightarrow{k_1}.\vec{x} + c_1\right)\right)\left(1 + \cos\left(\overrightarrow{k_2}.\vec{x} + c_2\right)\right)\left(1 + \cos\left(\overrightarrow{k_3}.\vec{x} + c_3\right)\right)$$

where A determines the peak firing rate ($8A$) and does not affect spatial correlations with $f(\vec{x})$. Each of the 900 ideal grids was a unique combination of one of 30 increments of scale and 30 increments of orientation. Orientation varied in increments of 2° between 0 and 60°, while scale varied in equal increments between the scale of the cell as estimated from the screening autocorrelogram ±20%. Spatial cross-correlograms between the ideal grid and the recorded ratemap were used to find the Pearson product-moment correlation at all spatial offsets, ignoring unvisited bins. From the spatial

cross-correlogram the phase offsets which yielded the highest correlation for that orientation and scale combination were then identified. The independent fit value was then calculated as the highest correlation found between the independent model and the ratemap across all combinations of scale, orientation and phase. The independent model was used to assess the scale and orientation of the recorded grid, with subsequently fitted 'local' and 'global' models constrained to the orientation and scale achieving the best fit in the independent model. Further, as the independent fit indicates the highest correlation that can be achieved between any ideal grid and the ratemap, grid cells with an independent fit <0.45 were excluded from subsequent analysis to account for cells which could not be well fitted by any model. A criterion of 0.45 was chosen as it was near the median independent fit of 0.4702. As such, just under half of grid cell/session conjunctions were excluded (65/144, 45.14%). Varying the required inclusion criterion between 0.4 and 0.5 had little effect on the overall conclusions drawn from the results.

To subsequently fit the local and global models two additional ideal grids were generated, each with the same orientation and scale as was found to achieve the best independent fit. In the local model the ideal grid had the same phase in the two compartments, whereas the global grid had a continuous phase across both compartments. The Pearson product-moment correlations between the firing rate map and the local and global ideal grids was then calculated at each offset as above. The maximum correlations achieved between the local and global grid and the recorded firing pattern were then identified. These maximum correlations were divided by the maximum correlation found under the independent fit, giving the local and global fit values, with which comparisons could be made across cells.

To assess the significance of the local and global fits, each recorded ratemap was also fitted with 1000 randomly phase offset grids. As in the local and global models each of the randomly offset grids was constrained to the same orientation and scale as was found under the independent model. However, each had a random phase offset (0-2π along the first two grid axes) between the two

compartments. For each of the 1000 grids a spatial cross-correlogram with the recorded ratemap was calculated to find the spatial offset which achieved the maximum correlation between that ideal grid and the ratemap. Again, as in the local and global model, the maximum correlation at any offset was divided by the independent fit to yield the fit value for each randomly offset grid. For each cell/session conjunction we then calculated the proportion of the 1000 randomly phase offset grids which achieved a fit greater than that of the local or global model. That is, we identified where the local and global fits fell in the distribution of fits achieved by the 1000 randomly offset grids. These values were then collapsed (mean averaged) within animals to create a single value for each animal in every session in which grid cells were recorded. Under the null hypothesis of no particular phase relationship between the grid firing in each compartment, a Wilcoxon signed rank test was used to examine whether the observed proportions of the 1000 grids with a better fit than the local or global model differed significantly from 0.5. This test was applied in the first and last five sessions.

To identify whether the apparent transition between local and global representations could be explained by biases in the sampling of different grid modules across time we repeated the above analyses on particular subsets of the data. First, the analysis was repeated including only those cells with a scale of 45-55cm, as estimated from the spatial autocorrelogram calculated from the screening environment's ratemap. In addition, the analysis was repeated on only those grid cells from a single animal in which a number of cells with independent fits > 0.45 and from a single module were recorded over a number of sessions. Grid cells were assigned to putative modules based on the scale of the cell in the screening environment. Concurrently recorded cells were considered to be of the same grid module if the ratio of the larger to the smaller scale cell was less than 1.4 [S6].

Finally, we fitted recorded grids in the same way as described above, except the thirds of each compartment closest to and furthest from the corridor were fitted separately. Again, for each recording we discarded the corridor to produce a ratemap comprising both compartments separated

by the gap due to the dividing walls. Here, we then divided the two compartments into three sections according to distance from the corridor to produce three ratemaps of ~180x30cm. Idealised grids were then fitted to the thirds closest to and furthest from the corridor for each cell under the same principles as above. Again, fits were collapsed within animals such that each data point corresponded to the average of all cells recorded from one animal in one session. These third-compartment fits were then used to test the hypothesis that the thirds of each compartment closest to the corridor are more globally coherent/less locally coherent than the thirds furthest away as they are closer together, such that there is reduced distance over which the path integrator may accumulate error. In the first five sessions, when the grids displayed a local representation, a paired, one-tailed t-test was used to ask whether the thirds furthest from the corridor were significantly more local than the thirds closest to the corridor. In contrast, in the last five sessions when the grids displayed a global representation, a paired, one-tailed t-test asked whether the grid patterns were more globally coherent in the thirds closest to rather than furthest from the corridor.

iv. Analysis of head direction cells

To analyse whether the activity of head direction cells disambiguated the two compartments, we calculated the angle of directional tuning and peak firing rate of head direction cells separately for the two compartments. Directional firing rate maps were calculated as described above, using positional and spiking data from either the left or right compartments. We then produced an angular cross-correlogram between the two directional firing rate maps for each cell by rotating one ratemap relative to the other and calculating the correlation between the two at each offset. To calculate the degree of angular offset between the directional tuning in each compartment we calculated the distance to the closest positive peak of the cross-correlogram from the origin, such that an offset of 0 indicates the highest correlation in the cross-correlogram occurred with no rotation of either directional ratemap. Angular offsets were then plotted as a function of experience of the multicompartment environment. The correlation between angular offset and recording

session was used to assess whether head direction cells disambiguated the two compartments with increasing experience. We next calculated the firing rate at the peak of directional tuning for the left and right compartments separately. A paired Wilcoxon signed-rank test was used to analyse whether head direction cell firing rates disambiguated the two compartments.

v. Phase offset analysis

The conclusions drawn from the fitting of ideal local and global grids were verified through a separate analysis of observed grid phase. In particular, we identified the difference in grid phase between the observed firing patterns in the right-hand compartment and that predicted from observed firing patterns in the left-hand compartment extended under a local or global representation. First, an ideal grid was fitted to the firing rate map recorded in the left-hand compartment of each grid cell according to the fitting principles described above. The ideal fitted grid was used to analyse the scale, orientation and phase of the recorded grid in the left compartment. Predictions were then made using the identified grid parameters about the location of grid fields in the right-hand compartment expected under a local or global firing pattern. That is, the local model predicts that firing fields replicate in the two compartments, so the phase in the right-hand compartment should equal the left. In the global model a single grid spans both compartments, thus the phase in the left compartment was 'projected' out to identify the phase in the right-hand compartment such that placed adjacent to one another a single grid spans both compartments. An ideal grid was then fitted to the firing pattern recorded in the right-hand compartment. Thus the identified phase of the recorded data could be compared to the phase predicted under the local and global models. The reported 'phase error' between the data and local or global models was then calculated as the magnitude of the vector connecting the observed and predicted phases in grid phase-space, and collapsed across all cells recorded in the same animal in the same session. Grid cells were again discarded where the previously calculated independent fit was <0.45. Further, the 10 data points where the predictions made by the local and global model

were most similar were also discarded to remove cells in which the models could not be distinguished. This equated to (18.52% of the 54 collapsed data points with an independent fit >0.45).

vi. Corridor grid cell classifier

The long and thin shape of the corridor meant analysis of the hexagonality of grid cell firing using a spatial autocorrelogram was unfeasible due to only sampling a small number of fields. To assess whether firing in the corridor was ‘grid-like’ we therefore used a grid cell classifier developed for analysing grid cells based on 1D linear-track firing [S5]. A smoothed firing rate map of the corridor of the multicompartment environment was first calculated. For each 2x2cm bin, a shuffled distribution of firing rates was calculated by shifting the positional information relative to spike times by at least 20 seconds and recalculating the firing rate in each bin for each of the 1000 shuffles. For each bin the proportion of shuffles which have a firing rate greater than that of the original firing rate map was then calculated. These proportions therefore allowed identification of bins in the original ratemap in which firing rates were higher or lower than would be expected by chance. Firing fields were defined as areas of at least 20 contiguous bins in which firing rates were greater than the 85th percentile of the bootstrapped shuffle distribution for those bins. Out of field regions were defined as areas with at least 20 contiguous bins where firing rates were less than the 5th percentile of the shuffled distribution. Cells were then classified as grid cells if: i. the corridor contained at least 3 firing fields as well as at least one out of field region. ii. 30% or more of the bins were assigned to either in-field or out-of-field areas. iii. The mean in-field to out-of-field firing rate ratio was greater than 2. The proportion of grid cells recorded in each day which satisfied these criteria was then plotted as a function of experience of the environment.

References:

S1. Barry, C., Hayman, R., Burgess, N. & Jeffery, K. J. (2007). Experience-dependent rescaling of entorhinal grids. *Nat. Neurosci.* **10,** 682–684.

S2. Kadir SN, Goodman DFM, Harris KD. (2014). High-dimensional cluster analysis with the masked EM algorithm. *Neural Computation* **26**:11.

S3. Sargolini, F., Fyhn, M., Hafting, T., McNaughton, B.L., Witter, M.P., Moser, M.-B., Moser, E.I. (2006). Conjunctive representation of position, direction, and velocity in entorhinal cortex. *Science* **312**, 758–762.

S4. Hafting, T., Fyhn, M., Bonnevie, T., Moser, M.-B., Moser, E.I. (2008). Hippocampus-independent phase precession in entorhinal grid cells. *Nature* **453** 1248-1242.

S5. Domnisoru, C., Kinkhabwala, A.A., Tank, D.W. (2013). Membrane potential dynamics of grid cells. *Nature* **495**, 199-204.

S6. Stensola, H., Stensola, T., Solstad, T., Froland, K., Moser, M.-B. & Moser, E.I. (2012). The entorhinal grid map is discretized. *Nature* **492** 72-78.
